# Supplementary figures and images for: Transforming RNA-Seq gene expression to track cancer progression in the multi-stage early to advanced-stage cancer development
Source: PLoS One. 2023 Apr 24;18(4):e0284458. doi: 10.1371/journal.pone.0284458 (PMC10124877; doi:10.1371/journal.pone.0284458)

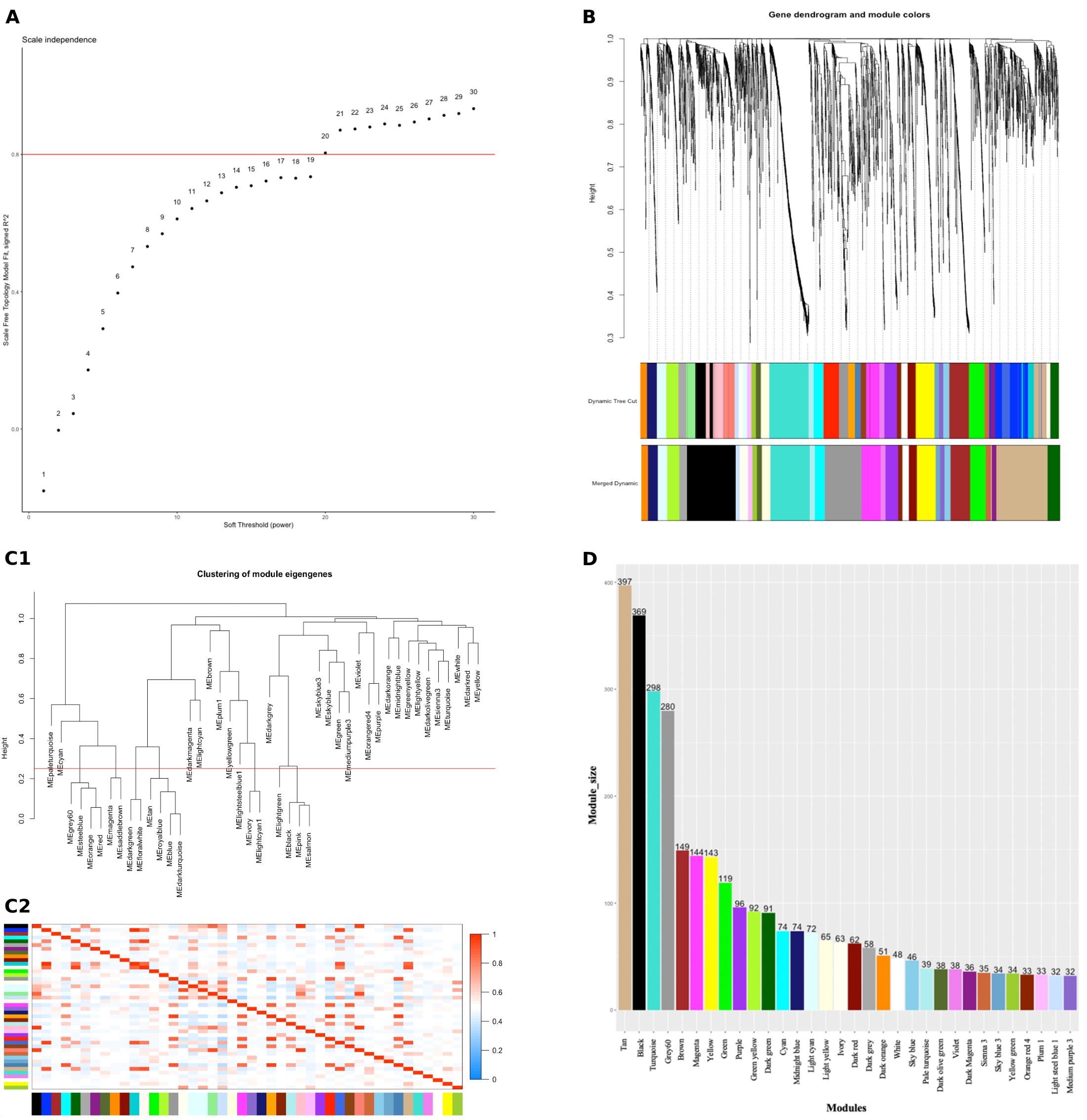

Supplement: S1 Fig — (A) Soft threshold power. (B) Gene clustering tree. Each colour underneath the dendrogram shows the module assignment, and branches above represent the genes. The dynamic tree cut shows the initial module detection and merged dynamic indicates the modules divided according to their similarity. (C1) Module eigengene dendrogram identified groups of correlated modules. The red line indicates the module eigengene threshold of 0.25 and (C2) Eigengene adjacency heatmap of different gene co-expression modules. In the heatmap, the blue colour represents low adjacency, while the red represents high adjacency. (D) Barplot of 32 co-expression modules constructed after similar modules were merged with module size at the top of each bar. (TIF) [file pone.0284458.s001.tif]

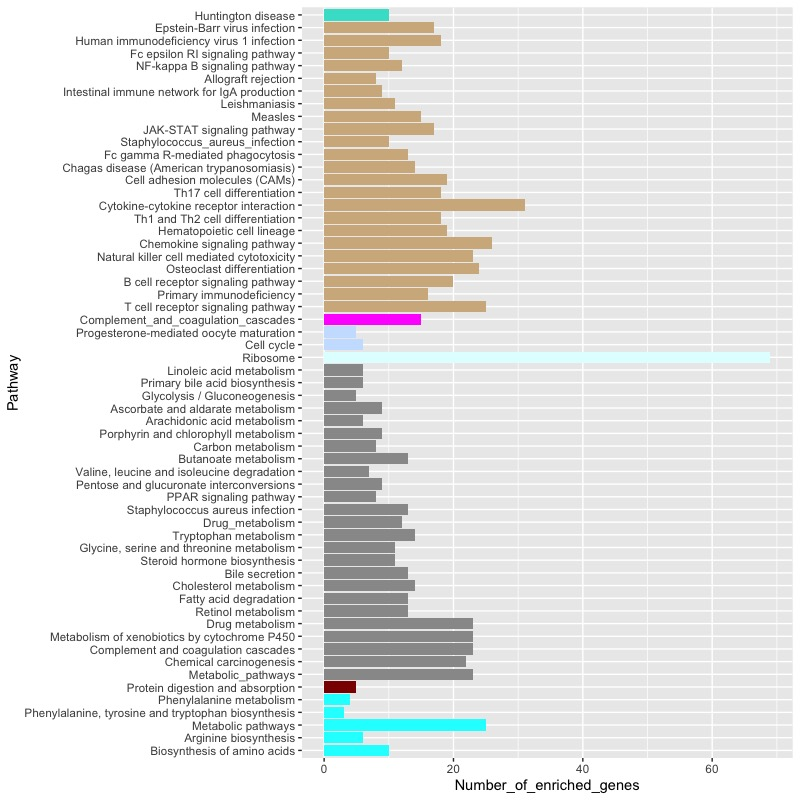

Supplement: S2 Fig — (TIF) [file pone.0284458.s002.tif]

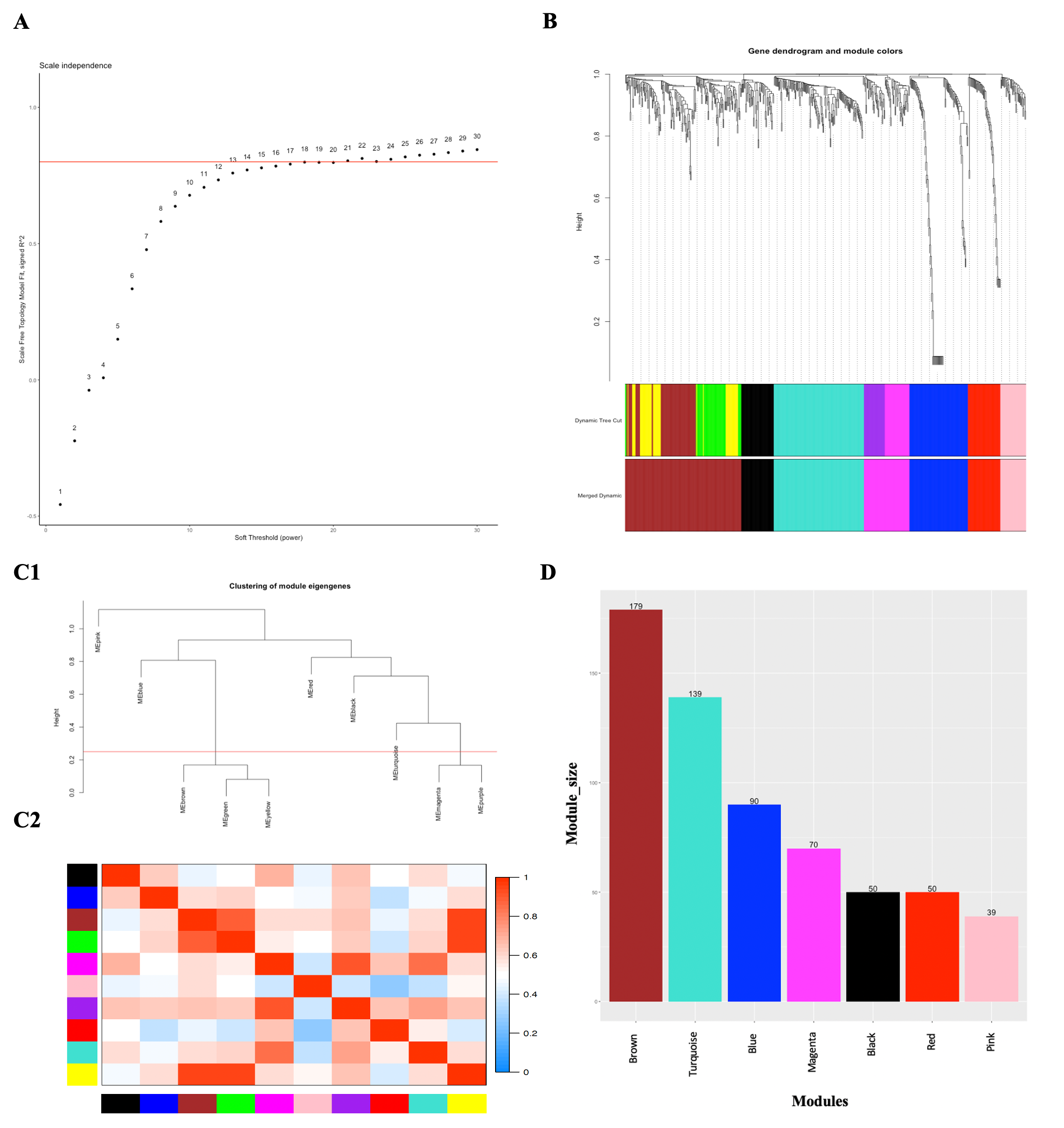

Supplement: S3 Fig — (A) Soft threshold power. (B) Gene clustering tree. Each colour underneath the dendrogram shows the module assignment, and branches above represent the genes. The dynamic tree cut shows the initial module detection and merged dynamic indicates the modules divided according to their similarity. (C1) Module eigengene dendrogram identified groups of correlated modules. The red line indicates the module eigengene threshold of 0.25 and (C2) Eigengene adjacency heatmap of different gene co-expression modules. In the heatmap, the blue colour represents low adjacency, while the red represents high adjacency. (D) Barplot of seven co-expression modules constructed after merged modules with module size at the top of each bar. (TIF) [file pone.0284458.s003.tif]

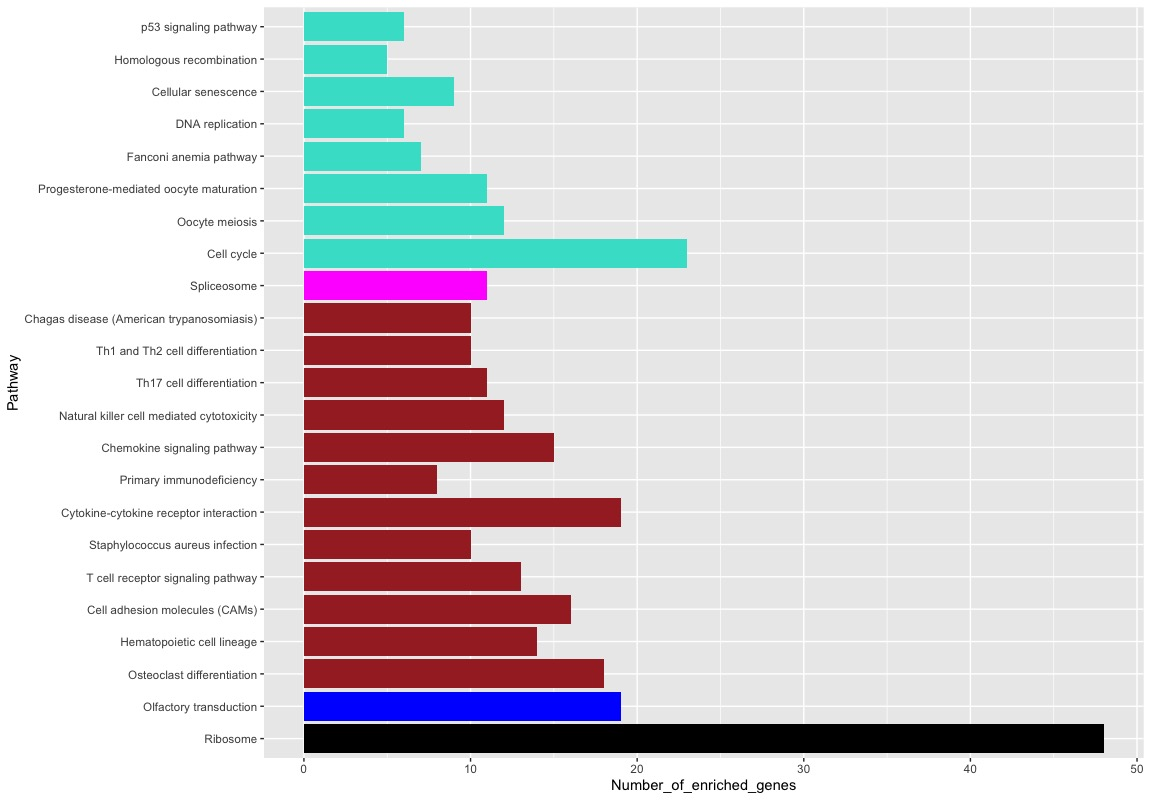

Supplement: S4 Fig — (TIF) [file pone.0284458.s004.tif]

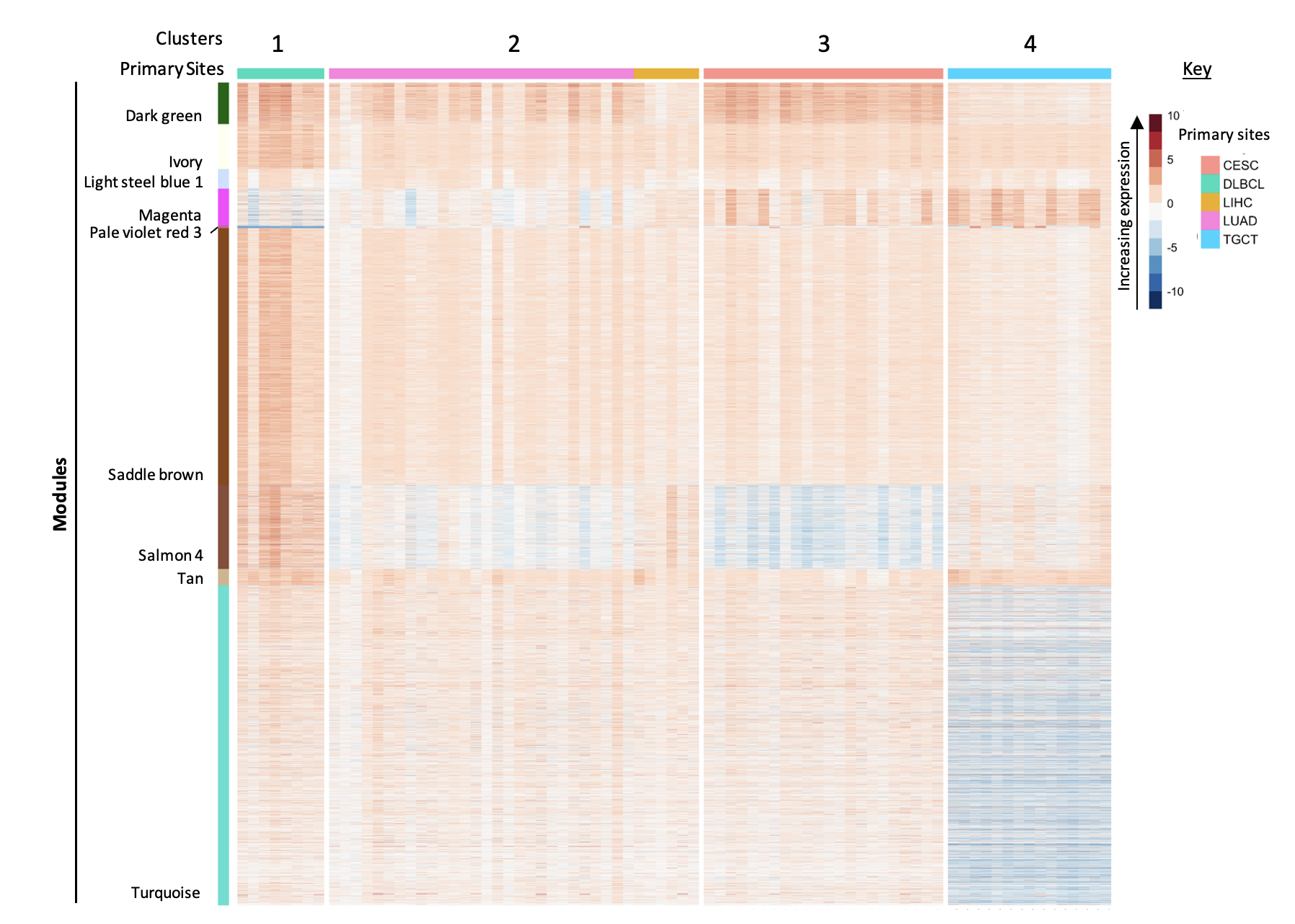

Supplement: S5 Fig — Normal tissue expression dataset was obtained from the Genotype-Tissue Expression (GTEx) Portal. To match the number of male/female ratios as in the late-stage cancer samples, the same number normal tissue samples of male/female ratios were randomly selected, except for cervical cancer, which only had 10 normal tissue samples. The colour bar on the left shows modules identified by WGCNA and enriched for functional pathway annotations. The rows are further composed of protein-coding genes with expression values obtained after data normalization. Clusters of similar cancer cohorts are indicated across the top and the cancer cohort are displayed by the colour bar along the top with the key on the right. *Primary sites abbreviations: CESC = Cervical squamous cell carcinoma; DLBCL = Diffuse Large B-cell Lymphoma; LIHC = Liver Hepatocellular Carcinoma; LUAD = Lung Adenocarcinoma; TGCT = Testicular Germ Cell Tumors. (TIF) [file pone.0284458.s005.tif]
