# Supplementary material for: Transforming RNA-Seq gene expression to track cancer progression in the multi-stage early to advanced-stage cancer development
Source: PLoS One. 2023 Apr 24;18(4):e0284458. doi: 10.1371/journal.pone.0284458 (PMC10124877; doi:10.1371/journal.pone.0284458)
Supplement: S1 File — (PDF) [file pone.0284458.s006.pdf]

S1 File: The data underlying the results presented in this study are publicly accessible from the UCSC Xena data browser (https://xenabrowser.net) from individual cancer cohorts.

(1) Datasets used in uncorrected and tissue-corrected analysis. The RNA-Seq gene expression and curated clinical public datasets that have been used: Large B-cell Lymphoma (DLBC), Liver Cancer (LIHC), Lung Adenocarcinoma (LUAD), Cervical Cancer (CESC), and Testicular Cancer (TGCT).

Cancer Name	Dataset ID	Dataset	Phenotypes
Large B-cell Lymphoma (DLBC)	TCGA-DLBC	https://gdc-hub.s3.us-east-1.amazonaws.com/download/TCGA-DLBC.htseq_counts.tsv.gz.	https://gdc-hub.s3.us-east-1.amazonaws.com/download/TCGA-DLBC.GDC_phenotype.tsv.gz.
Lung Adenocarcinoma (LUAD)	TCGA-LUAD	https://gdc-hub.s3.us-east-1.amazonaws.com/download/TCGA-LUAD.htseq_counts.tsv.gz.	https://gdc-hub.s3.us-east-1.amazonaws.com/download/TCGA-LUAD.GDC_phenotype.tsv.gz.
Liver Cancer (LIHC)	TCGA-LIHC	https://gdc-hub.s3.us-east-1.amazonaws.com/download/TCGA-LIHC.htseq_counts.tsv.gz.	https://gdc-hub.s3.us-east-1.amazonaws.com/download/TCGA-LIHC.GDC_phenotype.tsv.gz.
Cervical Cancer (CESC)	TCGA-CESC	https://gdc-hub.s3.us-east-1.amazonaws.com/download/TCGA-CESC.htseq_counts.tsv.gz.	https://gdc-hub.s3.us-east-1.amazonaws.com/download/TCGA-CESC.GDC_phenotype.tsv.gz.
Testicular Cancer (TGCT)	TCGA-TGCT	https://gdc-hub.s3.us-east-1.amazonaws.com/download/TCGA-TGCT.htseq_counts.tsv.gz.	https://gdc-hub.s3.us-east-1.amazonaws.com/download/TCGA-TGCT.GDC_phenotype.tsv.gz.

(2) Normal tissue expression dataset was obtained from the Genotype-Tissue Expression (GTEx) Portal. Dataset from the primary sites were extracted to match the individual cancer cohorts (last column).

Primary sites	Dataset ID	Dataset	Phenotypes	Matched Cancer
Whole Blood	GTEX	https://toil-xena-hub.s3.us-east-1.amazonaws.com/download/gtex_gene_expected_count.gz	https://toil-xena-hub.s3.us-east-1.amazonaws.com/download/GTEX_phenotype.gz	Large B-cell Lymphoma
Lung	GTEX	https://toil-xena-hub.s3.us-east-1.amazonaws.com/download/gtex_gene_expected_count.gz	https://toil-xena-hub.s3.us-east-1.amazonaws.com/download/GTEX_phenotype.gz	Lung Adenocarcinoma
Liver	GTEX	https://toil-xena-hub.s3.us-east-1.amazonaws.com/download/gtex_gene_expected_count.gz	https://toil-xena-hub.s3.us-east-1.amazonaws.com/download/GTEX_phenotype.gz	Liver Cancer
Cervix	GTEX	https://toil-xena-hub.s3.us-east-1.amazonaws.com/download/gtex_gene_expected_count.gz	https://toil-xena-hub.s3.us-east-1.amazonaws.com/download/GTEX_phenotype.gz	Cervical Cancer
Testis	GTEX	https://toil-xena-hub.s3.us-east-1.amazonaws.com/download/gtex_gene_expected_count.gz	https://toil-xena-hub.s3.us-east-1.amazonaws.com/download/GTEX_phenotype.gz	Testicular Cancer
