# Supplementary material for: Transforming RNA-Seq gene expression to track cancer progression in the multi-stage early to advanced-stage cancer development
Source: PLoS One. 2023 Apr 24;18(4):e0284458. doi: 10.1371/journal.pone.0284458 (PMC10124877; doi:10.1371/journal.pone.0284458)
Supplement: S2 File — The biological role indicates the role of the identified TF in cancer according to literature. (PDF) [file pone.0284458.s007.pdf]

S2 File: Top 5 TFs derived from the ChEA3 enrichment analysis of each tissue-corrected WGCNA module. The biological role indicates the role of the identified TF in cancer according to literature.

Module	Biological Role	TF	Overlapping genes	FDR
Black	FOX proteins are significantly implicated in cancer [1].	FOXB1	RPL5,RPL30,RPL32,RPL31,RPL34,RPLP0,RPL9,RPL7,RPS14,RPLP2,RPS10,RPL39,RPS13,RPL21,RPL23,RPS3A,RPL37A,RPL36A,RPS15A,RPS3,RPL15,RPL23A,RPS25,RPS27,RPS29,RPS20,RPS24,RPS23	1.61E-28
Black	Prognostic marker, high expression is unfavorable in liver cancer [2].	CHCHD3	RPL5,RPL30,RPL32,RPL31,RPL34,RPLP0,RPL10A,RPL9,RPL7,RPS14,RPLP2,RPS10,RPL39,RPS13,RPL21,RPL23,RPS3A,RPL37A,RPL36A,RPS3,RPL15,RPL23A,RPS25,RPS27,RPS29,RPS24,RPS23	2.14E-27
Black	Prognostic marker, high expression is unfavorable in liver cancer [3].	ZNF581	RPL5,RPL3,RPL32,RPLP0,RPL8,RPL10A,EEF1B2,RPL7A,RPS14,RPS18,RPLP2,RPS10,RPS13,RPS8,RPS6,RPSA,RPL27,RPL29,RPL12,RPS3,RPL14,RPL15,RPS23	5.41E-22
Black	Over expressed in various cancers, including hepatocellular carcinoma [4,5].	OTX1	RPL30,RPL31,RPL34,RPL9,RPL7,RPS14,RACK1,RPS10,RPL39,RPL21,RPL37A,RPL36A,RPS15A,RPL13,RPL15,RPS25,RPS27,RPS29,RPS24	9.13E-17
Black	Overexpression has been associated with the development of pancreatic [6,7], breast [8] and ovarian [9] cancers.	HES1	RPL30,RPL31,RPL34,RPL9,RPL7,RPS14,RPL39,RPL21,RPL23,RPL37A,RPL36A,RPL15,RPL23A,RPS27,RPS29,RPS20,RPS24,RPS23	1.2E-15
Brown	Down-regulated expression in hepatocellular carcinoma and gastric cancer [10,11].	IRF5	CD86,CD84,SPI1,CD80,LST1,ICAM3,CMKLR1,RNASE6,CYBB,MPEG1,OSCAR,TYROBP,BTK,CSF1R,IGSF6,FPR3,CORO1A,PIK3R5,SLAMF7,NCKAP1L,CD14,SLAMF1,CCR1,CD163,FAM78A,LY86,PILRA,ARHGAP30,FERMT3,SIGLEC9,ITGAM,PLEK,ITGB2,SIRPB2,SPN,HK3,FCGR3A,CD37,CCR5,CD53,FCER1G,NFAM1,FGR,HCK,MS4A6A,TLR8,LCP2,LCP1,PLEKHO2,DOCK2,SASH3,LILRA6,C1QA,WAS,LILRA2,AIF1,FGD2,CYTH4,LAIR1,LRRC25,IL	1.44E-67

			10RA,LAPTM5,LILRB1,LILRB2,LILRB3,LILRB4,CD4,SIGLEC1,MYO1F,C1QC	
Brown	Abnormal BATF expression in tumors predicted survival times of patients [12].<br><br>BATF expression could also predict immunotherapeutic and chemotherapy responses in cancer [12].	BATF	TRAF3IP3,CD80,SLA,IKZF1,SIT1,GPR171,TBC1D10C,CD96,TYROBP,ACAP1,CD8A,SP140,RASAL3,CORO1A,SAMSN1,PIK3R5,SLAMF7,LPXN,ICOS,SLAMF1,SH2D1A,NKG7,PILRA,PTPRC,ARHGAP30,CD27,FERMT3,ITK,PLEK,CD3G,CD3E,CD3D,GNGT2,CD37,CYTIP,CCR5,MAP4K1,CD53,IL16,APBB1IP,ZAP70,LCP2,LCP1,SASH3,CST7,CXCR3,CCL5,IL21R,TIGIT,S1PR4,P2RY10,TRAT1,IL10RA,LAPTM5,LILRB1,LILRB2,LILRB4,CD2,CD4,CD6,ABI3,CD5,IL2RB,CD7,SIGLEC1,PTPN7,CD247	1.03E-63
Brown	An increased incidence of TBX21 has been linked to cancer development [13,14].<br><br>TBX21 has been associated with poor prognosis in patients with lung adenocarcinoma [15].	TBX21	TRAF3IP3,SPI1,ICAM3,IKZF1,IL18RAP,SIT1,TBC1D10C,CD300A,DOK2,ACAP1,CD8A,RASAL3,CORO1A,SLAMF6,FAM78A,PYHIN1,NKG7,ARHGAP25,PTPRC,ARHGAP30,ITK,ITGAM,ITGB2,SIRPB2,ITGAL,CD3E,FCGR3A,TAGAP,CD37,TNFAIP8L2,CYTIP,MAP4K1,CD53,NFAM1,IL16,FGR,APBB1IP,HCK,ZAP70,TLR8,LCP2,LCP1,DOCK2,SASH3,LILRA6,WAS,AOAH,CST7,LILRA2,CYTH4,CCL5,S1PR4,LRRC25,P2RY13,IL10RA,LAPTM5,LILRB3,CD2,CD6,ABI3,IL2RB,CD7,MNDA,CD247,EVI2B,MYO1F	1.27E-62
Brown	Recent studies have reported significant functions of Arid5a in numerous types of cancer, including lung cancers [16-19].	ARID5A	CD86,SPI1,ICAM3,IKZF1,C3AR1,TBC1D10C,OSCAR,TYROBP,ACAP1,CSF1R,RASAL3,CORO1A,SAMSN1,PIK3R5,SLAMF7,LPXN,CD14,SLAMF1,CCR1,PILRA,ARHGAP25,ARHGAP30,FERMT3,SIGLEC9,PLEK,ITGB2,ITGAL,CD3E,TAGAP,CD37,CYTIP,MAP4K1,CD53,FCER1G,IL16,FGR,APBB1IP,HCK,ZAP70,LCP2,LCP1,PLEKHO2,DOCK2,SASH3,C1QA,WAS,CYTH4,IL21R,FCGR1A,S1PR4,IL10RA,L	2.81E-61

			APTM5,LILRB1,LILRB2,LILRB3,LILRB4,CD4,CD6,CD5,IL2RB,CD7,CD247,EVI2B,MYO1F,C1QC	
Brown	Significantly associated with cervical cancer prognosis [20].	SCML4	TRAF3IP3,GPR65,ICAM3,SLA,IKZF1,SLA2,SIT1,TBC1D10C,CD96,ACAP1,CD8A,ZNF831,RASAL3,CORO1A,LY9,TESPA1,LPXN,SLAMF6,ICOS,CD300LF,FAM78A,PYHIN1,SH2D1A,NKG7,ARHGAP25,PTPRC,ARHGAP30,CD27,ITK,CD3G,ITGAL,CD3E,CD3D,TNFSF13B,TAGAP,CD37,CYTIP,MAP4K1,CD53,IL16,APBB1IP,ZAP70,LCP2,LCP1,SASH3,WAS,CYTH4,CXCR3,CCL5,IL12RB1,S1PR4,P2RY10,TRAT1,IL10RA,SNX20,CD2,CD6,CD5,IL2RB,CD7,PTPN7,CD247,EVI2B,MYO1F	4.09E-60
Magenta	Affect TGF-β signaling to promote prostate cancer [21].	ZNF507	HNRNPU,XPO1,KPNB1,DHX9,IREB2,SRSF1,LARP1,TRA2B,NSD1,TRPM7,HNRNPA3,MGA,TJP1,HNRNPL	5.11E-9
Magenta	Prognostic marker, high expression is unfavorable in liver cancer [22].	ZNF207	TCERG1,ICE2,HNRNPU,XPO1,SRSF2,SRSF3,DHX9,IREB2,SRSF1,TRA2B,NSD1,TRPM7,NONO,MGA	5.11E-9
Magenta	SAFB protein levels predict poor prognosis of breast cancer patients [23].	SAFB	HNRNPU,RBM14,NCL,HNRNPH3,KPNB1,SF1,DHX9,SRSF1,GANAB,LARP1,HNRNPA3,NONO,HNRNPL,HNRNPD	5.11E-9
Magenta	These proteins have critical roles in development, differentiation, and tumorigenesis [24].	ZBTB39	HNRNPU,XPO1,GEMIN5,KPNB1,SF1,DHX9,SRSF1,LARP1,TRA2B,NSD1,HNRNPA3,NONO,HNRNPL,HNRNPD	5.11E-9
Magenta	CTCF has been identified as a putative driver gene in several cancer types [25].	CTCF	DDX46,HNRNPU,XPO1,NCL,KPNB1,SF1,DHX9,SRSF1,LARP1,NSD1,HNRNPA3,NONO,HNRNPL,HNRNPD	5.11E-9
Turquoise	E2F8 is correlated with the progression of cervical cancer [26].	E2F8	DSCC1,CCNF,HJURP,BUB1B,MKI67,CDC20,CHEK1,NUSAP1,OIP5,GTSE1,ESCO2,CDC25C,HASPIN,WDR76,CDC25A,SGO1,DEPDC1B,MELK,TIMELESS,KIF20A,CDCA2,PARPBP,CDCA3,TROAP,CDCA5,NCAPG,CDCA8,HMMR,PKMYT1,SKA3,IQGAP	1.35E-143

			3,NCAPH,RAD51AP1,CCNB2,CCNB1,ORC1,RACGAP1,CLSPN,FAM83D,FANCI,PLK4,STIL,PLK1,CDC6,NDC80,ZWINT,ANLN,TPX2,KIF18A,KIF18B,UBE2T,KIF4A,CDK1,TOP2A,ARHGAP11A,FEN1,NCAPG2,KIF14,MCM10,BRCA1,KIF11,FOXM1,LMNB1,KIF15,EXO1,NUF2,PBK,MYBL2,SPDL1,DLGAP5,CEP55,RFC4,CKAP2L,KIF23,CIP2A,CCNA2,ASPM,ESPL1,INCENP,KIFC1,DEPDC1,BIRC5,MCM4,KIF2C,MCM6,MTFR2,DTL,FAM72B,FAM72A,UHRF1,PRIM1,TTK,TYMS,AURKB,AURKA,CDC45,E2F2,RAD54L,BUB1,E2F7,GINS1,POLQ,CENPU,RRM2,SPAG5,SHCBP1,TICRR,CENPE,CENPF,CENPI,PRC1,TRIP13,CDKN3,MAD2L1	
Turquoise	Prognostic marker, high expression is unfavorable in liver cancer and lung [27,28].	CENPA	DSCC1,CCNF,HJURP,BUB1B,CDC20,CHEK1,NUSAP1,OIP5,NEK2,KPNA2,GTSE1,CDC25C,HASPIN,KNSTRN,CDC25A,SGO1,DEPDC1B,MELK,TIMELESS,KIF20A,PRR11,PIF1,CDCA2,PARPBP,CDCA3,TROAP,CDCA5,NCAPG,CDCA8,HMMR,PKMYT1,SKA3,IQGAP3,NCAPH,RAD51AP1,CCNB2,CCNB1,RACGAP1,FAM83D,FANCI,PLK4,STIL,UBE2C,PLK1,CDC6,NDC80,ZWINT,ANLN,TPX2,KIF18A,KIF18B,UBE2T,KIF4A,CDK1,TOP2A,ARHGAP11A,FEN1,NCAPG2,KIF14,MCM10,KIF11,FOXM1,LMNB1,KIF15,EXO1,NUF2,PBK,MYBL2,SPDL1,DLGAP5,CEP55,CKAP2L,KIF23,CIP2A,CCNA2,ASPM,ESPL1,INCENP,KIFC1,DEPDC1,BIRC5,MCM4,KIF2C,MTFR2,DTL,FAM72B,FAM72A,UHRF1,TTK,TYMS,AURKB,AURKA,CDC45,RAD54L,BUB1,GINS1,CENPU,RRM2,SPAG5,SHCBP1,TICRR,CENPE,CENPF,RAD51,PRC1,TRIP13,CDKN3,MAD2L1	7.77E-134

Turquoise	E2F7 promotes cell proliferation and metastasis in lung adenocarcinoma, liver cancer and head and neck cancer [29-31].	E2F7	DSCC1,CCNF,HJURP,BUB1B,MKI67,CDC20,NUSAP1,GTSE1,HASPIN,CDC25A,MELK,TIMELESS,KIF20A,CDCA2,CDCA5,NCAPG,CDCA8,HMMR,PKMYT1,SKA3,IQGAP3,NCAPH,RAD51AP1,CCNB1,ORC1,RACGAP1,CLSPN,FANCI,PLK4,STIL,PLK1,CDC6,NDC80,ZWINT,ANLN,TPX2,KIF18B,UBE2T,KIF4A,CDK1,TOP2A,ARHGAP11A,FEN1,NCAPG2,KIF14,MCM10,KIF11,FOXM1,LMNB1,KIF15,EXO1,NUF2,PBK,MYBL2,SPDL1,DLGAP5,CEP55,CKAP2L,KIF23,CIP2A,CCNA2,ASPM,ESPL1,INCENP,KIFC1,DEPDC1,BIRC5,MCM4,KIF2C,DTL,UHRF1,TTK,TYMS,AURKB,AURKA,RAD54L,BUB1,GINS1,POLQ,CENPU,RRM2,SPAG5,SHCBP1,TICRR,CENPE,CENPF,PRC1,TRIP13,MAD2L1	2.21E-103
Turquoise	E2F2 plays a significant role in tumor progression [32].	E2F2	CCNF,HJURP,MKI67,CHEK1,NUSAP1,GTSE1,ESCO2,HASPIN,WDR76,CDC25A,TIMELESS,TROAP,CDCA5,NCAPG,CDCA8,PKMYT1,NCAPH,SKA1,ORC1,CLSPN,FANCI,PLK4,STIL,CDC6,NDC80,ZWINT,KIF18B,ARHGAP11A,FEN1,NCAPG2,KIF14,MCM10,BRCA1,KIF11,FOXM1,LMNB1,KIF15,CHAF1B,EXO1,MYBL2,CKAP2L,CCNA2,ASPM,ESPL1,INCENP,KIFC1,MCM4,KIF2C,DTL,UHRF1,TYMS,CDC45,RAD54L,GINS1,POLQ,CENPU,RRM2,SPAG5,SHCBP1,TICRR,PRC1,CENPK,SPC24	2.49E-64
Turquoise	FOXN4 can be used as candidate prognostic biomarkers for lung adenocarcinoma [33].	FOXN4	CCNF,HJURP,BUB1B,CDC20,NUSAP1,NEK2,KPNA2,GTSE1,CDC25C,KNSTRN,CDC25A,SGO1,DEPDC1B,MELK,KIF20A,PIF1,CDCA2,CDCA3,TROAP,NCAPG,CDCA8,IQGAP3,NCAPH,CCNB2,CCNB1,RACGAP1,PLK4,UBE2C,PLK1,NDC80,TPX2,KIF18A,UBE2T,KIF4A,CDK1,TOP2A,KIF14,MCM10,BRCA1,KIF11,KIF15,NUF2,PBK,DLGAP5,CKAP2L,KIF23,CIP2A,CCNA2,ASPM,ESPL1,KIFC1,BIRC5,KI	2.49E-64

			F2C,FAM72B,TTK,AURKB,BUB1,E2F7,SPAG5,CENPE,CENPF,PRC1,CDKN3	

S2 File References:

1. Bach DH, Long NP, Luu TT, Anh NH, Kwon SW, Lee SK. The Dominant Role of Forkhead Box Proteins in Cancer. Int J Mol Sci. 2018 Oct 22;19(10):3279. doi: 10.3390/ijms19103279. PMID: 30360388; PMCID: PMC6213973.

2. The human protein atlas (HPA) [Internet]. Human Pathology Atlas [cited 2023 Jan 9]: CHCHD3 gene available from: https://www.proteinatlas.org/ENSG00000106554-CHCHD3/pathology/liver+cancer.

3. The human protein atlas (HPA) [Internet]. Human Pathology Atlas [cited 2023 Jan 9]: ZNF581 gene available from: https://www.proteinatlas.org/ENSG00000171425-ZNF581/pathology/liver+cancer.

4. de Haas T, Oussoren E, Grajkowska W, et al. OTX1 and OTX2 expression correlates with the clinicopathologic classification of medulloblastomas. J Neuropathol Exp Neurol. 2006 Feb;65(2):176-86. doi: 10.1097/01.jnen.0000199576.70923.8a. PMID: 16462208.

5. Terrinoni A, Pagani IS, Zucchi I, et al. OTX1 expression in breast cancer is regulated by p53. Oncogene. 2011 Jul 7;30(27):3096-103. doi: 10.1038/onc.2011.31.

6. Jensen J, Pedersen EE, Galante P, Hald J, Heller RS, Ishibashi M, et al. Control of endodermal endocrine development by Hes-1. Nature genetics. 2000 Jan;24(1):36-44. doi: 10.1038/71657. PMID: 10615124.

7. Katoh M, Katoh M. Integrative genomic analyses on HES/HEY family: Notch-independent HES1, HES3 transcription in undifferentiated ES cells, and Notch-dependent HES1, HES5, HEY1, HEY2, HEYL transcription in fetal tissues, adult tissues, or cancer. Int J Oncol. 2007 Aug;31(2):461-6. PMID: 17611704.

8. Farnie G, Clarke RB, Spence K, Pinnock N, Brennan K, Anderson NG, et al. Novel cell culture technique for primary ductal carcinoma in situ: role of Notch and epidermal growth factor receptor signaling pathways. J Natl Cancer Inst. 2007 Apr 18;99(8):616-27. doi: 10.1093/jnci/djk133. PMID: 17440163.

9. Wang X, Fu Y, Chen X, Ye J, Lu B, Ye F, et al. The expressions of bHLH gene HES1 and HES5 in advanced ovarian serous adenocarcinomas and their prognostic significance: a retrospective clinical study. J Cancer Res Clin Oncol. 2010 Jul;136(7):989-96. doi: 10.1007/s00432-009-0744-8. Epub 2009 Dec 20. PMID: 20091184; PMCID: PMC2874490.

10. Shin SH, Kim BH, Jang JJ, Suh KS, Kang GH. Identification of novel methylation markers in hepatocellular carcinoma using a methylation array. J Korean Med Sci. 2010 Aug;25(8):1152-9. doi: 10.3346/jkms.2010.25.8.1152. Epub 2010 Jul 20. PMID: 20676325; PMCID: PMC2908783.

11. Yamashita M, Toyota M, Suzuki H, Nojima M, Yamamoto E, Kamimae S, et al. DNA methylation of interferon regulatory factors in gastric cancer and noncancerous gastric mucosae. Cancer Sci. 2010 Jul;101(7):1708-16. doi: 10.1111/j.1349-7006.2010.01581.x. Epub 2010 Apr 2. PMID: 20507321.

12. Jia C, Ma Y, Wang M, Liu W, Tang F, Chen J. Evidence of Omics, Immune Infiltration, and Pharmacogenomics for BATF in a Pan-Cancer Cohort. Front Mol Biosci. 2022 Apr 29;9:844721. doi: 10.3389/fmolb.2022.844721. PMID: 35573731; PMCID: PMC9098817.

13. Yu H, Yang J, Jiao S, Li Y, Zhang W, Wang J. T-box transcription factor 21 expression in breast cancer and its relationship with prognosis. Int J Clin Exp Pathol. 2014 Sep 15;7(10):6906-13. PMID: 25400774; PMCID: PMC4230155.

14. Lin ZW, Wu LX, Xie Y, Ou X, Tian PK, Liu XP, et al. The expression levels of transcription factors T-bet, GATA-3, RORγt and FOXP3 in peripheral blood lymphocyte (PBL) of patients with liver cancer and their significance. Int J Med Sci. 2015 Jan 1;12(1):7-16. doi: 10.7150/ijms.8352. PMID: 25552913; PMCID: PMC4278870.

15. Zhao S, Shen W, Yu J, Wang L. TBX21 predicts prognosis of patients and drives cancer stem cell maintenance via the TBX21-IL-4 pathway in lung adenocarcinoma. Stem Cell Res Ther. 2018 Apr 3;9(1):89. doi: 10.1186/s13287-018-0820-6. PMID: 29615105; PMCID: PMC5883886.

16. Sarode P, Zheng X, Giotopoulou GA, Weigert A, Kuenne C, Günther S, et al. Reprogramming of tumor-associated macrophages by targeting β-catenin/FOSL2/ARID5A signaling: A potential treatment of lung cancer. Sci Adv. 2020 Jun 5;6(23):eaaz6105. doi: 10.1126/sciadv.aaz6105. PMID: 32548260; PMCID: PMC7274802.

17. Zhou Q, Zhou J, Fan J. Expression and Prognostic Value of ARID5A and its Correlation With Tumor-Infiltrating Immune Cells in Glioma. Front Oncol. 2021 May 19;11:638803. doi: 10.3389/fonc.2021.638803. PMID: 34094918; PMCID: PMC8172138.

18. Parajuli G, Tekguc M, Wing JB, Hashimoto A, Okuzaki D, Hirata T, et al. Arid5a Promotes Immune Evasion by Augmenting Tryptophan Metabolism and Chemokine Expression. Cancer Immunol Res. 2021 Aug;9(8):862-876. doi: 10.1158/2326-6066.CIR-21-0014. Epub 2021 May 18. PMID: 34006522.

19. Zhang J, Hou S, You Z, Li G, Xu S, Li X, Zhang X, Lei B, Pang D. Expression and prognostic values of ARID family members in breast cancer. Aging (Albany NY). 2021 Feb 11;13(4):5621-5637. doi: 10.18632/aging.202489. Epub 2021 Feb 11. PMID: 33592583; PMCID: PMC7950271.

20. Peng-Qiang Zhong, Xing-Xing Yan, Wei-Jia Wang, MengZhi Hong, Peisong Chen, Min Liu. Identification and Validation of LYZ and CCL19 as Prognostic Genes in the Cervical Cancer Micro-Environment. Clin. Exp. Obstet. Gynecol. 2022; 49(6), 144. https://doi.org/10.31083/j.ceog4906144.

21. Kwon W, Choi SK, Kim D, Kim HG, Park JK, Han JE, et al. ZNF507 affects TGF-β signaling via TGFBR1 and MAP3K8 activation in the progression of prostate cancer to an aggressive state. J Exp Clin Cancer Res. 2021 Sep 18;40(1):291. doi: 10.1186/s13046-021-02094-3. PMID: 34537073; PMCID: PMC8449443.

22. The human protein atlas (HPA) [Internet]. Human Pathology Atlas [cited 2023 Jan 9]: ZNF207 gene available from: https://www.proteinatlas.org/ENSG00000010244-ZNF207/pathology/liver+cancer.

23. Hammerich-Hille S, Bardout VJ, Hilsenbeck SG, Osborne CK, Oesterreich S. Low SAFB levels are associated with worse outcome in breast cancer patients. Breast Cancer Res Treat. 2010 Jun;121(2):503-9. doi: 10.1007/s10549-008-0297-6. Epub 2009 Jan 10. PMID: 19137425.

24. Lee SU, Maeda T. POK/ZBTB proteins: an emerging family of proteins that regulate lymphoid development and function. Immunol Rev. 2012 May;247(1):107-19. doi: 10.1111/j.1600-065X.2012.01116.x. PMID: 22500835; PMCID: PMC3334328.

25. Marshall AD, Bailey CG, Champ K, Vellozzi M, O'Young P, Metierre C, et al. CTCF genetic alterations in endometrial carcinoma are pro-tumorigenic. Oncogene. 2017 Jul 20;36(29):4100-4110. doi: 10.1038/onc.2017.25. Epub 2017 Mar 20. PMID: 28319062; PMCID: PMC5519450.

26. Kim LK, Park SA, Eoh KJ, Heo TH, Kim YT, Kim HJ. E2F8 regulates the proliferation and invasion through epithelial-mesenchymal transition in cervical cancer. Int J Biol Sci. 2020 Jan 1;16(2):320-329. doi: 10.7150/ijbs.37686. PMID: 31929759; PMCID: PMC6949145.

27. The human protein atlas (HPA) [Internet]. Human Pathology Atlas [cited 2023 Jan 9]: CENPA gene available from: https://www.proteinatlas.org/ENSG00000115163-CENPA/pathology/liver+cancer.

28. The human protein atlas (HPA) [Internet]. Human Pathology Atlas [cited 2023 Jan 9]: CENPA gene available from: https://www.proteinatlas.org/ENSG00000115163-CENPA/pathology/lung+cancer.

29. Liang R, Xiao G, Wang M, Li X, Li Y, Hui Z, et al. SNHG6 functions as a competing endogenous RNA to regulate E2F7 expression by sponging miR-26a-5p in lung adenocarcinoma. Biomed Pharmacother. 2018 Nov;107:1434-1446. doi: 10.1016/j.biopha.2018.08.099. Epub 2018 Sep 1. PMID: 30257360.

30. Ma YS, Lv ZW, Yu F, Chang ZY, Cong XL, Zhong XM, et al. MicroRNA-302a/d inhibits the self-renewal capability and cell cycle entry of liver cancer stem cells by targeting the E2F7/AKT axis. J Exp Clin Cancer Res. 2018 Oct 16;37(1):252. doi: 10.1186/s13046-018-0927-8. PMID: 30326936; PMCID: PMC6192354.

31. Saleh AD, Cheng H, Martin SE, Si H, Ormanoglu P, Carlson S, et al. Integrated Genomic and Functional microRNA Analysis Identifies miR-30-5p as a Tumor Suppressor and Potential Therapeutic Nanomedicine in Head and Neck Cancer. Clin Cancer Res. 2019 May 1;25(9):2860-2873. doi: 10.1158/1078-0432.CCR-18-0716. PMID: 30723145; PMCID: PMC6497577.

32. Shen S, Wang Y. Expression and Prognostic Role of E2F2 in Hepatocellular Carcinoma. Int J Gen Med. 2021 Nov 18;14:8463-8472. doi: 10.2147/IJGM.S334033. PMID: 34824545; PMCID: PMC8609201.

33. Yao Y, Zhang T, Qi L, Liu R, Liu G, Li J, Sun C. Identification of Four Genes as Prognosis Signatures in Lung Adenocarcinoma Microenvironment. Pharmgenomics Pers Med. 2021 Jan 8;14:15-26. doi: 10.2147/PGPM.S283414. PMID: 33447073; PMCID: PMC7802904.
