# Supplementary material for: Transforming RNA-Seq gene expression to track cancer progression in the multi-stage early to advanced-stage cancer development
Source: PLoS One. 2023 Apr 24;18(4):e0284458. doi: 10.1371/journal.pone.0284458 (PMC10124877; doi:10.1371/journal.pone.0284458)
Supplement: S3 File — A list of TFs and their corresponding rank according to ARCHS4 co-expression, with documented information about their biological function associated with survival in the context of cervical cancer. The genes in bold were previously found [55] to play a role in cervical cancer survival. (PDF) [file pone.0284458.s008.pdf]

S3 File: Transcription factors (TFs) enrichment analysis of tissue-corrected WGCNA brown module. A list of TFs and their corresponding rank according to ARCHS4 co-expression, with documented information about their biological function associated with survival in the context of cervical cancer. The genes in bold were previously found [55] to play a role in cervical cancer survival.

Survival associated with TF	Rank	TF	Overlapping genes	FDR
Significantly associated with cervical cancer prognosis [1].	5	SCML4	**MAP4K1**,TRAF3IP3,GPR65,ICAM3,SLA,IKZF1,SLA2,SIT1,**TBC1D10C**,CD96,**ACAP1**,CD8A,**ZNF831**,**RASAL3**,CORO1A,LY9,TESPA1,LPXN,SLAMF6,ICOS,CD300LF,FAM78A,PYHIN1,**SH2D1A**,NKG7,ARHGAP25,PTPRC,ARHGAP30,CD27,ITK,CD3G,ITGAL,CD3E,CD3D,TNFSF13B,TAGAP,CD37,CYTIP,CD53,IL16,APBB1IP,ZAP70,LCP2,LCP1,SASH3,WAS,CYTH4,CXCR3,CCL5,IL12RB1,S1PR4,**P2RY10**,TRAT1,IL10RA,SNX20,CD2,CD6,CD5,IL2RB,CD7,PTPN7,CD247,EVI2B,MYO1F	4.09E-60
Prognostic marker, high expression is favorable in cervical cancer [2].	9	SNAI3	**MAP4K1**,TRAF3IP3,SPI1,LST1,ICAM3,**TBC1D10C**,CD300A,OSCAR,TYROBP,**ACAP1**,IGSF6,**RASAL3**,CORO1A,PIK3R5,CCR1,FAM78A,PILRA,ARHGAP25,ARHGAP30,FERMT3,SIGLEC9,ITGAM,ITGB2,SIRPB2,ITGAL,CD3E,HK3,FCGR3A,CD37,TNFAIP8L2,CD53,NFAM1,IL16,FGR,APBB1IP,HCK,TLR8,LCP2,LCP1,PLEKHO2,SASH3,LILRA6,WAS,LILRA1,AOAH,LILRA2,CYTH4,S1PR4,LRRC25,P2RY13,IL10RA,LAPTM5,LILRB2,LILRB3,CD4,ABI3,CD7,MNDA,CD247,EVI2B,MYO1F	3.1E-56
Prognostic marker, high expression is favorable in cervical cancer [3].	14	IKZF1	**MAP4K1**,TRAF3IP3,ICAM3,GPR174,**TBC1D10C**,MPEG1,**ACAP1**,**RASAL3**,CORO1A,PIK3R5,NCKAP1L,FAM78A,ARHGAP25,PTPRC,ARHGAP30,FERMT3,ITK,ITGB2,SIRPB2,ITGAL,CD3E,PIK3CG,SPN,TAGAP,CD37,CYTIP,CCR2,CD53,NFAM1,IL16,FGR,APBB1IP,HCK,ZAP70,TLR8,LCP2,LCP1,DOCK2,SASH3,WAS,LILRA1,AOAH,CYTH4,S1PR4,LRRC25,P2RY13,IL10RA,LAPTM5,CD4,CD6,CD5,IL2RB,CD7,MNDA,CD247,EVI2B,MYO1F	3.13E-51
Prognostic marker, high expression is favorable in cervical cancer [4].	24	IKZF3	**MAP4K1**,TRAF3IP3,ICAM3,IKZF1,GPR174,SIT1,**GPR171**,**TBC1D10C**,PRKCB,**ACAP1**,CD8A,SP140,**RASAL3**,CORO1A,LY9,NCKAP1L,SLAMF6,FAM78A,PYHIN1,NKG7,ARHGAP25,PTPRC,ARHGAP30,	1.41E-47

			ITK,CD3G,ITGAL,CD3E,CD3D,SPN,TAGAP,CD37,CYTIP,CD53,FCRL3,IL16,APBB1IP,ZAP70,LCP2,LCP1,DOCK2,SASH3,IL21R,IL12RB1,TIGIT,**P2RY10**,IL10RA,LAPTM5,SCIMP,CD6,IL2RB,CD7,**PDCD1**,CD247,EVI2B	
Prognostic marker, high expression is favorable in cervical cancer [5].	34	FOXP3	**MAP4K1**,TRAF3IP3,ICAM3,SLA,IKZF1,GPR174,SIT1,**TBC1D10C**,UBASH3A,**ACAP1**,SP140,**RASAL3**,CORO1A,LPXN,ICOS,FAM78A,ARHGAP25,PTPRC,ARHGAP30,CD27,ITK,ITGAL,CD3E,CD3D,TAGAP,CD37,CYTIP,CD53,IL16,APBB1IP,ZAP70,LCP2,LCP1,DOCK2,SASH3,WAS,CYTH4,CXCR3,TIGIT,S1PR4,**P2RY10**,TRAT1,IL10RA,CD2,CD4,CD6,CD5,IL2RB,CD7,CD247	1.19E-42
May serve as a tumor suppressor gene in cervical cancer [6].	49	RUNX3	**MAP4K1**,TRAF3IP3,ICAM3,IKZF1,IL18RAP,**TBC1D10C**,**ACAP1**,**RASAL3**,CORO1A,PIK3R5,NCKAP1L,SLAMF1,FAM78A,ARHGAP25,PTPRC,ARHGAP30,ITGB2,ITGAL,CD3E,SPN,CD37,CYTIP,CD53,IL16,FGR,APBB1IP,ZAP70,IFNG,LCP2,LCP1,DOCK2,SASH3,WAS,CYTH4,TBX21,IL21R,S1PR4,**P2RY10**,IL10RA,LAPTM5,CD6,IL2RB,CD7,CD247,EVI2B,MYO1F	8.23E-38
High ETS1 levels exhibit a poorer prognosis than those with low ETS1 levels in cervical cancer [7,8].	59	ETS1	**MAP4K1**,TRAF3IP3,ICAM3,IKZF1,GPR174,**TBC1D10C**,**ACAP1**,**ZNF831**,**RASAL3**,CORO1A,NCKAP1L,SLAMF6,FAM78A,ARHGAP25,PTPRC,ARHGAP30,ITK,ITGB2,ITGAL,CD3E,TAGAP,CD37,CYTIP,CD53,IL16,APBB1IP,ZAP70,LCP2,LCP1,DOCK2,SASH3,WAS,CYTH4,S1PR4,**P2RY10**,IL10RA,LAPTM5,CD6,CD5,IL2RB,CD7,CD247,EVI2B	2.44E-34
Low expression is associated with poor prognosis in cervical cancer [9].	136	IRF4	**MAP4K1**,CD80,IKZF1,**GPR171**,SP140,**RASAL3**,LPXN,ICOS,SLAMF1,ARHGAP30,ITK,SPN,CYTIP,APBB1IP,IFNG,LCP1,DOCK2,SASH3,IL21R,**P2RY10**,LILRB1,SCIMP,CD6,IL2RB,CD7	4.34E-15
Prognostic marker, high expression is favorable in cervical cancer [10].	157	ZNF266	MAP4K1,IKZF1,**TBC1D10C**,**ACAP1**,RASAL3,LY9,ICOS,PYHIN1,PTPRC,CD3G,CD37,CYTIP,FCRL3,IL16,APBB1IP,ZAP70,IL12RB1,CD6,EVI2B	9.94E-10

S3 File References:

1. Peng-Qiang Zhong, Xing-Xing Yan, Wei-Jia Wang, MengZhi Hong, Peisong Chen, Min Liu. Identification and Validation of LYZ and CCL19 as Prognostic Genes in the Cervical Cancer Micro-Environment. Clin. Exp. Obstet. Gynecol. 2022; 49(6), 144. https://doi.org/10.31083/j.ceog4906144.

2. The human protein atlas (HPA) [Internet]. Human Pathology Atlas [cited 2023 Jan 10]: SNAI3 gene available from: https://www.proteinatlas.org/ENSG00000185669-SNAI3/pathology/cervical+cancer.

3. The human protein atlas (HPA) [Internet]. Human Pathology Atlas [cited 2023 Jan 10]: IKZF1 gene available from: https://www.proteinatlas.org/ENSG00000185811-IKZF1/pathology/cervical+cancer.

4. The human protein atlas (HPA) [Internet]. Human Pathology Atlas [cited 2023 Jan 10]: IKZF3 gene available from: https://www.proteinatlas.org/ENSG00000161405-IKZF3/pathology/cervical+cancer.

5. The human protein atlas (HPA) [Internet]. Human Pathology Atlas [cited 2023 Jan 10]: FOXP3 gene available from: https://www.proteinatlas.org/ENSG00000049768-FOXP3/pathology/cervical+cancer.

6. Li Z, Fan P, Deng M, Zeng C. The roles of RUNX3 in cervical cancer cells *in vitro*. Oncol Lett. 2018 Jun;15(6):8729-8734. doi: 10.3892/ol.2018.8419.

7. Xu FL, Li YL, Wang ZD, Feng YJ. Expression and significance about VEGF, KDR, MMP-1, and transcription factor Ets-1 in human cervical carcinoma. Zhongguo Yi Xue Ke Xue Yuan Xue Bao. 2003 Aug;25(4):396-400. Chinese. PMID: 12974081.

8. Fujimoto J, Aoki I, Toyoki H, Khatun S, Tamaya T. Clinical implications of expression of ETS-1 related to angiogenesis in uterine cervical cancers. Ann Oncol. 2002 Oct;13(10):1598-604. doi: 10.1093/annonc/mdf248. PMID: 12377648.

9. Deng ZM, Dai FF, Zhou Q, Cheng YX. Hsa_circ_0000301 facilitates the progression of cervical cancer by targeting miR-1228-3p/IRF4 Axis. BMC Cancer. 2021 May 21;21(1):583. doi: 10.1186/s12885-021-08331-4. PMID: 34020619; PMCID: PMC8140416.

10. The human protein atlas (HPA) [Internet]. Human Pathology Atlas [cited 2023 Jan 10]: ZNF266 gene available from: https://www.proteinatlas.org/ENSG00000174652-ZNF266/pathology/cervical+cancer.
