# Supplementary material for: Transforming RNA-Seq gene expression to track cancer progression in the multi-stage early to advanced-stage cancer development
Source: PLoS One. 2023 Apr 24;18(4):e0284458. doi: 10.1371/journal.pone.0284458 (PMC10124877; doi:10.1371/journal.pone.0284458)
Supplement: Attachment — Submitted filename: Response to Reviewers.pdf [file pone.0284458.s010.pdf]

6. Review Comments to the Author

Reviewer #1: The authors have addressed satisfactorily my comments and I appreciate that the authors made their code available.

Reviewer #2: The paper describes application of a previously published method proposed ("tissue-corrected" normalization approach of reference 4) to the comparison of samples from early vs late stages of cancer. Applying an existing method in a somewhat different context does not amount to a new methodological development.

Although the computational formulas used in the manuscript were the same as a previously published method, cited in reference 4, the variables that were applied to the computational formulas in this manuscript were unique and led to novel findings. The approach of the computational formulas, in the previously published paper, used cancer samples normalized with normal tissue samples, whereas our approach normalized late-stage samples with early-stage samples with the concept of tracking cancer progression and identifying new cancer subtypes, which led to substantially different results. Thus our approach can be considered as a different method. We illustrated in the supplementary of the revised manuscript that the approach used in reference 4, showed that clustering only allowed for the segregation of distinct cancer types, which is in concordance with the objective of the original method (S3 Fig). While our approach and concept for tracking cancer progression allowed for an in-depth clustering based on the distinct cancer types as well as clusters (subtypes) within cancer types. The discovery of cancer subtypes will have a significant impact in the field of cancer biology and precision medicine research, as our approach enables the tracking of cancer progression, that in turn allows for the development of more effective treatment strategies, aids in patients' welfare, and improve prognosis.

My few main concerns have not been fully addressed in the revised manuscript.

1. This manuscript does not include any benchmarking, apart from showing an interesting but anecdotal consistency with previously reported biological results, even in the revised version, which describes a biologically meaningful clustering of 12 PDCD1 associated genes. A more rigorous large-scale methodological evaluation and comparison with other computational approaches would be important.

The editor and another reviewer recommended highlighting the advantage of the proposed computational method and its contrast with the one presented in reference 4. Therefore, we benchmarked our approach in the revised manuscript by including the outcome of late-stage cancer samples normalized with normal tissue samples in the supplementary (S3 Fig).

We illustrate below the advantage of our approach in contrast to the original approach for the computational formulas. First, the unnormalized gene expression shows two heterogeneous cancer clusters (Fig 1A of this document). Second, the approach cited in reference 4, where cancer samples are normalized with normal samples and clustering allowed for the segregation between distinct cancer types, however, it failed to provide in-depth clustering based on clusters (subtypes) within cancer types (Fig 1B of this document). The approach used in the manuscript in which late-stage samples were normalized with early-stage samples shows in-depth clustering based on clusters (subtypes) within cancer types (Fig 1C of this document). The discovery of the eight segregated clusters (cancer subtypes) was validated by matching the clinical and prognostic features to the clusters. As a result, the variations in gene expression, such as in cervical cancer that was associated with survival were stratified by our approach but not by the approach of normalizing with normal samples. Additionally, we included citations from the literature to give more credibility to our results. Therefore, the approach and concept to the computational formulas in this manuscript provided an increased power in differentiating samples based on cancer type and clinical and phenotypic information which contributes to the scientific community.

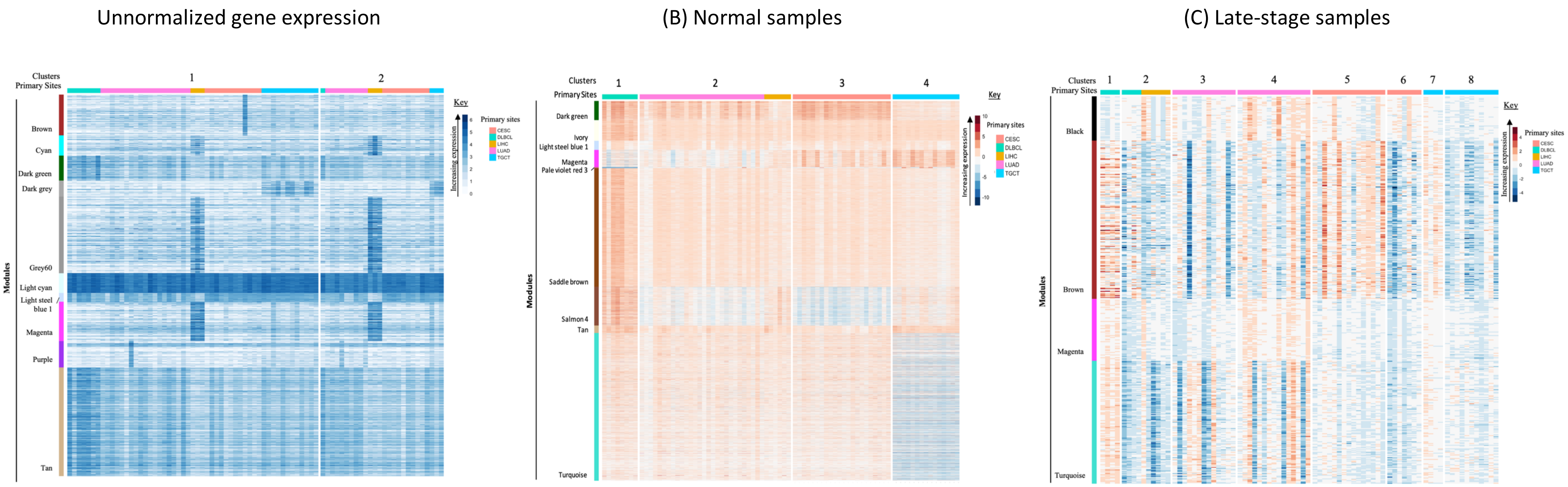

Fig 1: Benchmark and comparison of the computational method. The normalization of cancer samples with late-stage cancer samples (right) provided a more in-depth clustering based on the distinct cancer types as well as cluster (subtypes) within cancer types.

2. To show the value of the method to potential users, in addition to valuable examples of confirming previously known gene associations, it would be important to illustrate how this method can help generate new hypotheses of previously uncharacterized biological associations or mechanisms.

The development of high-throughput genome analysis techniques for research on cancer subtypes has a significant impact on the analysis and clinical treatment of multiple cancers. Therefore, the discovery of cancer subtypes is useful for guiding clinical treatment of multiple cancers.

The value of this method thus aids with hypotheses that seek to explore various novel cancer subtypes that segregate by different gene expression profiles and further investigate the biological association, clinical, or prognostic features linked to the cancer subtypes (clusters). Additionally, hypotheses that investigate cancer progression and identify cancer subtypes with different progression. New users can further use this method to find new subtypes in their data and associate it with the clinical data that they have.

This above information was further added to the manuscript.

Based on the authors' response about the rationale for using a small dataset focused on a particular cancer type, this manuscript almost seems a pilot to a bigger project focused on HIV effects on Diffuse Large-B cell Lymphoma. This bigger project, hopefully with a larger patient cohort, larger body of results and hopefully new mechanistic hypotheses, may warrant a publication in this journal.

Due to the high cost and limited funding for whole-genome sequencing and RNA-Seq, the research that focuses on HIV effects on DLBCL described in the first response to reviewers document will have a limited sample number. It should be noted that these few samples will only contribute to DLBCL and will not have an impact on the significant findings made in the multiple cancers that were evaluated in this study. Therefore, to validate the findings in the manuscript, the computational method was applied to multiple cancers. This demonstrates that the computational method enables for the discovery of cancer subtypes in multiple cancers and not restricted to a specific cancer type, and thus vital to the wider scientific community.

Minor comments:

1. My question about the interaction of cancer-specific signatures and autoimmune responses was not addressed by the authors: Would autoimmune signatures contribute as an unrelated confounding factor to late-stage cancer expression signatures?

Unfortunately, the clinical data did not provide information about the patients' autoimmune disease status. Therefore, it is highly improbable that the cluster segregation observed is related to autoimmune diseases.

2. "Therefore, the use of the WikiPathway database was sufficient in providing the knowledge we required in the context of this specific investigation."

Using multiple databases of functional gene categories in addition to WikiPathways would be important, especially in the context of this manuscript where functional enrichment is a major source of confirming that results are reasonable. Some of these additional databases include more mechanistic knowledge, e.g. empirically validated TF targets, which would be beneficial for generating new mechanistic hypotheses based on the method's results.

Thank you for the insightful feedback. We agree, it will be beneficial to include an additional enrichment analysis. Therefore, we first performed a Transcription factor (TF) enrichment analysis using ChEA3 database web-server application, with the implementation of the genes of interest that were grouped inside each module found by WGCNA, to assess TF activity yielding new biological insights. The top 5 TF entries were supplemented with the revised manuscript (S2 File). The documentation also confirms, with supported literature, several TF relationships with the cancers evaluated in this study.

Additionally, all TF entries of the brown module were analysed to offer a better understanding of the associations between the observed gene expression changes and TFs in the context of cervical cancer, as this module allowed for the segregation of two clusters associated with survival. The TFs that were linked with the MAP4K1 gene in which the TF was found to effect cervical cancer survival was extracted and included in the revised manuscript (S3 File). Several

co-expressed genes that also play a role in cervical cancer survival identified by Kannan et al. were also linked to the TFs and are highlighted (S3 File).

Secondly, KEGG enrichment analysis was performed, and found that the tissue-corrected WGCNA gene modules were cancer-related, while several uncorrected WGCNA gene modules showed repetition of the same pathway. This indicates that the uncorrected dataset, which did not undergo normalization, did not efficiently depict gene expression differences. Hence, we found that the conclusions drawn using both WikiPathways and KEGG were similar and resembled relatedness of the enriched genes.

The results of KEGG pathway enrichment analysis for both uncorrected and tissue-corrected WGCNA modules are included below (Fig 2 & Fig 3 of this document). Please advise if it would be beneficial to include in the manuscript?

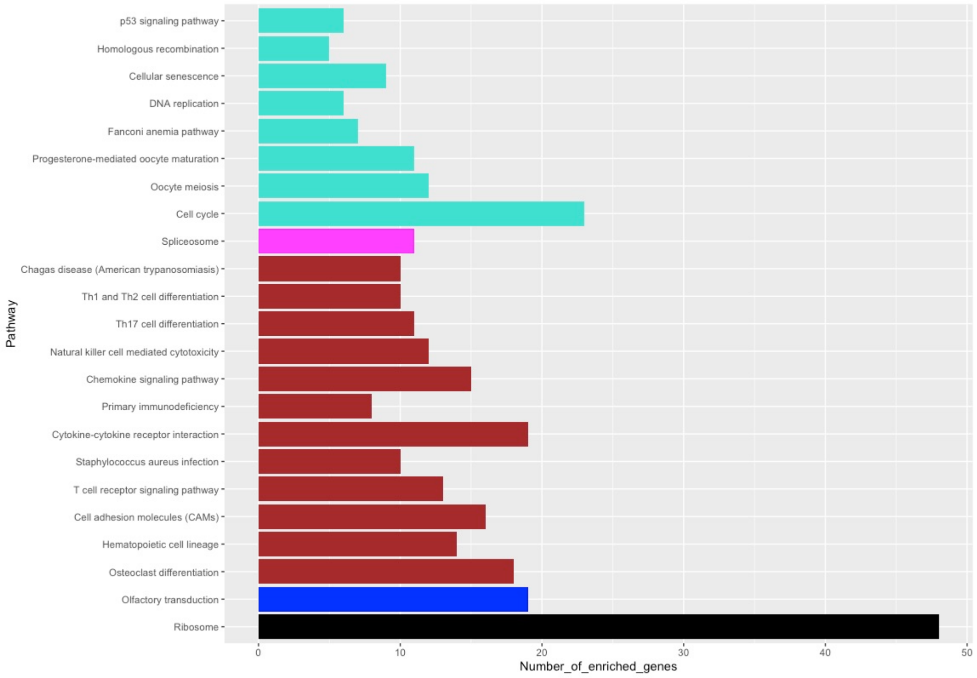

Fig 2: KEGG enrichment of gene modules detected by WGCNA from the tissue-corrected RNA dataset using the ORA, WebGestalt.

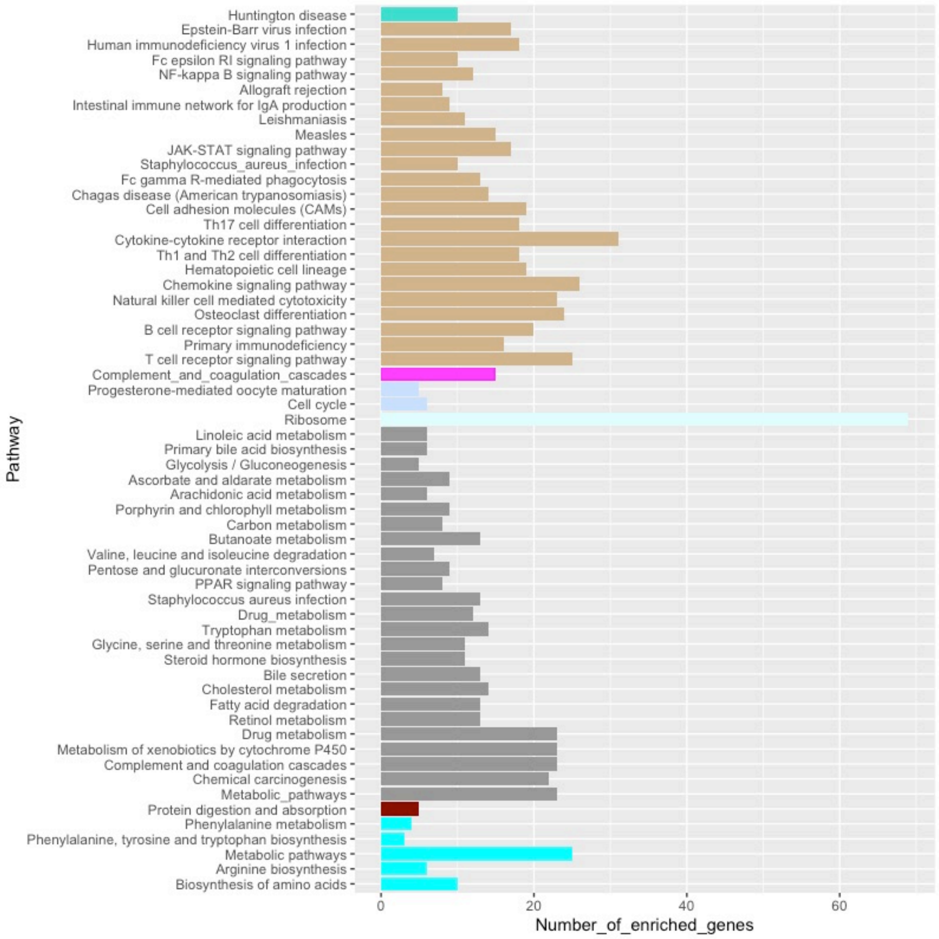

Fig 3: KEGG enrichment of gene modules detected by WGCNA from the uncorrected RNA dataset using the ORA, WebGestalt.

References:

1. Kannan S, O'Connor GM, Bakker EY. Molecular Mechanisms of PD-1 and PD-L1 Activity on a Pan-Cancer Basis: A Bioinformatic Exploratory Study. Int J Mol Sci. 2021 May 22;22(11):5478. doi: 10.3390/ijms22115478. PMID: 34067485; PMCID: PMC8196980.
