# Supplementary material for: Transforming RNA-Seq gene expression to track cancer progression in the multi-stage early to advanced-stage cancer development
Source: PLoS One. 2023 Apr 24;18(4):e0284458. doi: 10.1371/journal.pone.0284458 (PMC10124877; doi:10.1371/journal.pone.0284458)
Supplement: Attachment — Submitted filename: Response to Reviewers.pdf [file pone.0284458.s011.pdf]

Review Comments to the Author

Reviewer #1: Overall I think the manuscript lacks some clarity. I have the following comments:

- Figure 2 is not needed in my opinion. The point that it makes is that the same pathways were enriched in several modules, this could just be mentioned in the text.

Thank you for your comment. Figure 2 has been removed from the revised manuscript and the revised manuscript reads that the same pathways were enriched in several modules.

- In line 75-82 the use of the word normalized is confusing. The authors give an introduction to RNA-seq and where they mention that raw counts need to be normalized to be used for further analysis. I do agree but normalizing raw counts reads is the normal standard practice, although in a different way in which authors propose normalization in this work. The paper in which they base their normalization method actually uses TPM which is a normalization strategy for RNA-seq. I think the authors need to address why they used unnormalized values (raw reads) as input instead of TPMs, and also point out in the introduction that they are proposing a normalization strategy that does not replace standard normalization but it is specific to their research question. Furthermore, comparing unnormalized gene expression of different samples makes no sense. For figure 1 they would have to explain that even normalized expression values such as tpm would still not address the issue depicted in Figure 1. The authors here propose normalization of gene expression for late stage cancer samples using early stage cancer samples. I understand what the author means but then need to clarify that their normalization method is not the same kind of normalization done to RNA-seq raw counts data such as accounting for library size.

The title of the manuscript has been amended to "Transforming RNA-Seq gene expression to track cancer progression in the multi-stage early to advanced-stage cancer development". Further, the introduction sentence (line 75 - 82) which referred to the term "normalization" and "unnormalized " was amended to "transformed" and "raw", respectively. This is to prevent confusion about the term 'normalization', as the method in the manuscript was not used for differential gene expression analysis, but to track cancer progression and identify new cancer subtypes.

It has been reported that transcriptome-profiling strictly using TPM has been misused (Zhao et al., 2020). Zhao et al. stated that it is a common misconception that TPM values are already normalized, and thus should be comparable across samples or RNA-Seq projects. However, this is unfortunately not always true (Zhao et al., 2020). This statement was supported by their review in which they found that TPM is easily influenced by sample preparation protocols and should not be used to compare between different tissue parts as we investigated in our study. Hence, TPM normalizes transcript levels within samples and thus the direct comparison of TPM across samples is problematic. Also, it was found that the differences in TPM values would be due to the experimental protocol and not biologically relevant (Zhao et al., 2020). Therefore, raw data was used to retain the biological meaningful expression between different genes and across conditions.

It has been further found that raw RNA-Seq data seems to have the potential to decipher pathological status better than when the data is normalized (Han & Men, 2018). The same study found that some normalization approaches (e.g RPKM) bring negative effects in disease diagnosis. Their results also suggest that normalized data may not demonstrate statistically significant advantages in disease diagnosis than its raw form (Han & Men, 2018). Therefore, we decided to not use TPM expression values and used raw counts to retain the biological meaningful expression between different genes and samples. We included in the methodology section (line 140 in the revised manuscript) the following sentence to rational the use of raw count RNA-Seq data; "raw data may perform better for capturing more original transcriptome patterns in different disease conditions (Han & Men, 2018)".

- In line 244 the use of the word implemented is inaccurate. In computer science "implementing" implies writing code, not just executing or running a program with your own dataset.

In order to clarify the ambiguity regarding the term 'implemented', the sentence has been amended in the revised manuscript as follows: "Transcription factor (TF) enrichment analysis was performed on the genes of interest that were grouped inside each module found by WGCNA using the ChEA3 database web-server application."

- Most of the p values are not represented in a standard way (missing zeros before decimal points among others).

Thank you for your comment, we have included the zeros before the decimal points.

- I don't think screened is the appropriate verb here (line 360).

Thank you for your comment. We have revised the term "screened" and chosen a more suitable verb. Thus the sentence has been amended (line 363 in the revised manuscript) and reads as follows: "The tissue-corrected protein-coding genes were inserted into WGCNA."

Accordingly, identical occurrences have been amended in the same manner as above in lines 304, 766, and 781.

- Not sure what the next phase menas: "It was also found that the brown module has the highest enrichment". Enrichment score?, number of enriched pathways? pathways with the most genes?

We recognize that the phrase does raise questions, thus we provided more context and clarification with regard to the highest enrichment in the revised manuscript. The amended sentence in the revised manuscript reads as follows: "It was also found that the brown module has the highest total genes detected in biological pathways"

Accordingly, an identical occurrences in line 318 have been amended in the same manner as above.

- Modules can be enriched with certain pathways, but genes are not enriched, therefore all instances of "enriched genes" should be corrected.

Thank you very much. We agree that enrichment is performed on a set of genes, therefore, we have changed the occurrence of "enriched genes' and only refer to the genes.

- Line 401 "The WGCNA module genes were further subjected to TF" -> TF enrichment analysis

Thank you for the comment, the amendment has been made and now reflects ‘TF enrichment analysis’ (line 406 in the revised manuscript).

- "Moreover, they showed that PDCD1 significantly co-expressed with the following 15 genes, whose high expression is beneficial for cervical cancer patients; MAP4K1, ACAP1, CST7, CXCR6, GPR171, GZMH, GZMK, P2RY10, RASAL3, SH2D1A, TBC1D10C, ZNF831, GZMM, JAKMIP1, and PSTPIP1 [54]." Here the reference is a link to the human protein atlas for MAP4K1, so the reference only refers to the first gene and it seems like the authors claim it is for all 15 genes. If it is the case that they tried all 15 genes maybe they should include those figures as supplementary, for all 15 genes, or further explain in the text that they tested the genes in a website. As it currently stands readers expect to find a reference to a paper that claims that high expression of all 15 genes are beneficial for cervical cancer patients which is not what the reference is.

Two references have been added to the revised manuscript. Therefore, reference 54 referred to in the above section has been moved down to reference 56 (line 756 in the revised manuscript), which is linked to a paper by Kannan et al., 2021.

- In line 483 there is a claim that more than one tool was utilized for TF enrichment analyisis and I believe only one was used.

Thank you for this comment. Only one tool was utilized for TF enrichment analysis and therefore the sentence was amended and the revised manuscript refers to an established computation tool.

Reviewer #2: The authors addressed most of my comments in this new revision. As a response to the authors' question in the rebuttal, I suggest that functional pathway enrichment analyses based on KEGG database (Fig. 2 and 3 of rebuttal) should be included in the manuscript as supplementary figure(s). This is a minor recommendation that I trust the authors to follow through.

Thank you for responding to our question and providing valuable input. We have included the functional pathway enrichment analysis based on KEGG database in the revised manuscript as part of the methodology section (line 242) and included the results as supplementary figures(s).

References of response to reviewers document:

Han H, Men K. How does normalization impact RNA-seq disease diagnosis? J Biomed Inform. 2018 Sep;85:80-92. doi: 10.1016/j.jbi.2018.07.016. Epub 2018 Jul 21. PMID: 30041017.

Zhao S, Ye Z, Stanton R. Misuse of RPKM or TPM normalization when comparing across samples and sequencing protocols. RNA. 2020 Aug;26(8):903-909. doi: 10.1261/rna.074922.120. Epub 2020 Apr 13. PMID: 32284352; PMCID: PMC7373998.
