# Supplementary material for: Transforming RNA-Seq gene expression to track cancer progression in the multi-stage early to advanced-stage cancer development
Source: PLoS One. 2023 Apr 24;18(4):e0284458. doi: 10.1371/journal.pone.0284458 (PMC10124877; doi:10.1371/journal.pone.0284458)
Supplement: Attachment — Submitted filename: Response to Reviewers.docx [file pone.0284458.s009.docx]

Editor comments:

1. Particularly relevant is to highlight the advantages of your proposed method and its contrast with the one presented in reference 4, as well as the biological relevance of your findings so that the full extent of your contribution will be clearer.

To highlight the advantage of our normalization method, we included the result of late-stage cancer samples normalized with normal tissue samples as a supplement to the manuscript (kindly refer to the response to question 1.6). It should be noted that the normalized gene expression profiles using normal tissue samples, and clustering allowed for the segregation between distinct cancer types (S3 Fig), however, it failed to provide in-depth clustering based on cluster (subtypes) within cancer types. As a result, the variations in gene expression, such as in cervical cancer that was associated with survival, could not be stratified by normalizing late-stage cancer samples with normal tissue.

Additionally, a recent study by Kannan et al., 2021 focused on PDCD1 (a gene typically related to its expression on tumor-infiltrating lymphocytes). Their study found 15 co-expressed genes, whose high expression is beneficial for cervical cancer patients, this included the MAP4K1 gene that allowed for the segregation of Clusters 5 and 6 (Fig 5 in the revised manuscript) based on its prognostic value (Kindly refer to the response to question 2.1.b.). We compared their finding to the results of our normalization method and discovered the PDCD1 gene as well as 12 of the 15 genes co-expressed within the brown module. Therefore, the results obtained with our method by normalizing late-stage with early-stage cancer samples demonstrate the ability of the method to cluster samples by cancer progression.

2. Journal Requirements: When submitting your revision, we need you to address these additional requirements.

Please ensure that your manuscript meets PLOS ONE's style requirements, including those for file naming. The PLOS ONE style templates can be found at

https://journals.plos.org/plosone/s/file?id=wjVg/PLOSOne_formatting_sample_main_body.pdf and

https://journals.plos.org/plosone/s/file?id=ba62/PLOSOne_formatting_sample_title_authors_affiliations.pdf

To our knowledge we followed the PLOS ONE’s style requirements as provided in the templates. Kindly let us know if we should make any amendments.

**Reviewer #1:** The authors show that by normalizing the gene expression from late stage cancer using early stage cancer samples to track progression. The normalization method has been applied before using non tumor samples to normalize instead of early stage samples as in this case.

Minor suggestions/comments:

1.1 Figure 1 could include depiction of foldchange sizes in the two cancer types.

In Figure 1, we found that cancer type 1 showed a gene expression fold increase of 4 from early to advanced-stage cancer. Cancer type 2 showed a gene expression fold increase of 1 from early to advanced-stage cancer.

We included the foldchange sizes in the Figure 1 description as follows. **Fig 1. Non-normalized RNA-Seq data of advanced-stage and early-stage gene expression of gene *x* in two cancer types.** Cancer type 1 and cancer type 2 show a gene expression fold increase of 4 and 1, respectively, from early to advanced-stage cancer.

1.2. Most, if not all, of the subfigures in Figure 2 should be supplementary figures, sames as with Figure 5.

We agree, all the subfigures in Figure 2 and Figure 5 were added as supplementary figures. Kindly see S1 and S2 Fig in the revised manuscript. Accordingly, all figure numbering has been amended for figure labels and the in-text reference to the figures.

1.3. In the methods section, in the vector multiplication it needs to be clarified which operation is being applied; the notation implies cross product, but probably the one being used is element-wise multiplication (Hadamard product).

Thank you for your valued comment. We agree, the method used is element-wise multiplication (Hadamard product). Therefore, we changed the notation “×” (cross product) to $⨀$ (Hadamard product).

Equation 1 and 2 has been amended as follows: X*_i_* = P^A^*_I_* $⨀$ A*_i_* (equation 1) and Y*_i_* = P^E^*_I_* $⨀$ E*_i_* (equation 2).

1.4. Including the code for normalization and analysis steps would improve the paper, it would clarify steps, and would be of use to the commuity. The code could be included as either supplementary or with a github (or similar link.

Yes, we agree. We included a github code link for the normalization in the methods section. Link: <https://github.com/3270006/tracking-cancer-progression>.

In the readme, we included a link for the clustering that we used the scripts from on github: https://github.com/fgfrost/PanCan_RNA-Seq.

1.5. An explanation of the rational for the differential expression steps would be very useful. Why not use a standard differential expression analysis package such as DeSEQ2 or EdgeR?

Thank you for your comment. We understand that the title may have caused confusion in relation to a differential gene expression (DGE) analysis, as it referred to the identification of differentially expressed RNA-Seq, therefore the title were changed accordingly to: “A normalization method to track cancer progression with RNA-Seq gene expression in the multi-stage early to advanced-stage cancer development”. We apologize and highlight the aim of the normalization method in the manuscript was to track cancer progression from early to late-stage and correct for genes that display less expression variability in advanced-stage cancer samples but display a high variability in early-stage cancer samples. Therefore, the objective was to use the computation model and predict cancer clusters (subtypes) that progressed differently and/or similarly. Clinical data were used to find clinical and prognostic features that match the clusters segregation. For example, for clusters 5 and 6 (Fig 5 in the revised manuscript) the normalization method allowed for cervical cancer samples to segregate based on survival risk.

Generally, a standard DGE analysis between early and late-stage cancer samples will result in a list of differentially expressed genes (DEGs) specific to the two groups (early and late-stage). Therefore, clustering operation of the cancer samples based on the identified DEGs will systematically subject the samples to segregate into early and late-stage clusters, which was not the aim of the investigation. The results of the normalization method used in the manuscript were able to segregate clusters between and within a cancer based on features in late-stage cancer samples.

1.6. It would be interesting to know if using early stage works like a surrogate of normal tissue (as in the original publication for the normalization method) or it is actually better or gives a different insight.

Thank you for your valuable comment. We agree that it will be more beneficial to show that the use of early-stage samples provided a better insight than using normal tissue. Therefore, as a supplement to this manuscript, we provided the results of late-stage cancer samples normalized with normal tissue samples. We found that normalizing late-stage cancer samples with early-stage cancer samples, and clustering the normalized expression samples provided a better understanding of clinical and phenotypic patterns of patient samples within and between the same cancer type.

The following were added to the discussion in the manuscript: “To further demonstrate the significance of late-stage cancer samples normalized with early-stage cancer samples, an investigation was carried out with a normal tissue expression dataset from the Genotype-Tissue Expression (GTEx) Portal (GTEx Consortium, 2017). Normalized gene expression profiles using normal tissue samples were clustered and allowed for the segregation between distinct cancer types (S3 Fig). However, it failed to provide in-depth clustering based on subtypes within cancer types. As a result, the variations in gene expression, such as in cervical cancer that was associated with survival, could not be stratified by normalizing late-stage cancer samples with normal tissue. The results obtained with our method by normalizing late-stage with early-stage cancer samples demonstrate the ability of the method to cluster samples by cancer progression, rather than simply by cancer type as with the use of normal samples.”

Reviewer #2: The manuscript by Livesey et al describes a computational method aimed at the identification of gene expression signatures associated with cancer development from early to advanced stages. A central approach is to analyze multiple cancer types from multiple tissue sites, normalize all expression values for each gene at advanced cancer stage by the average expression value at the early stage, and apply the WGCNA method to identify gene modules corresponding to cancer progression. This method is a modification of a previously described approach (reference 4) to the identification of gene expression signatures in various combined cancer types compared to normal tissue samples. The method proposed in this manuscript is methodologically identical to the “tissue-corrected” normalization approach of reference 4, only applied to the comparison of samples from early vs late stages of cancer. The authors describe the application of their method to publicly available gene expression data from early and late stages of five cancer types, with 4 to 28 samples in each group. They compared the WGCNA results based on original expression data to the results based on the normalized data and observed a stronger pathway enrichment in WGCNA gene modules and a more clinically relevant clustering by sample type based on the normalized data. The manuscript is clearly written, with the exception of a few minor passages. My general concerns are the novelty of the proposed methodology, the evaluation approach, and a very minimal discussion of the actual known and new gene signatures detected by this method.

Major comments:

2.1. The direct advantage of this method to the discovery of known and new markers of advanced cancer stages is not described to a full extent. Detecting MAP4K1 (Fig. 8,9) whose upregulation is known to be associated with better outcomes is an interesting finding.

2.1.a. Could this finding be made by a more basic differential expression analysis between early and late stages of cervical cancer?

Thank you for your comment. We understand that the title may have caused confusion in relation to a DGE analysis, as it referred to the identification of differentially expressed RNA-Seq, therefore the title were changed accordingly to: “A normalization method to track cancer progression with RNA-Seq gene expression in the multi-stage early to advanced-stage cancer development”. We apologize and highlight the aim of the manuscript was to track cancer progression from early to late-stages and correct for genes that display less expression variability in advanced-stage cancer samples but display a high variability in early-stage cancer samples. Therefore, the objective was to use the computation model and predict cancer clusters (subtypes) that progressed differently and/or similarly. Clinical data were used to find clinical and prognostic features that match the clusters segregation. For example, for clusters 5 and 6 (Fig 5 in the revised manuscript) the normalization method allowed for cervical cancer samples to segregate based on survival risk.

The finding of MAP4K1 in cervical cancer patients was to demonstrate that the normalization was successful and the biological information was preserved. Therefore, the aim was achieved in that we were able to investigate cancer progression through the RNA-Seq expression profiles across the multi-stage process of cancer development. This discovery also served to highlight the potential of using this normalization method for future disease analysis to identify and validate biomarkers in the development of cancer.

We continued to conduct a basic DGE analysis using DESeq2 on cervical cancer samples, with early and late-stages serving as the two risk groups. Applying a threshold of α = 0.05 and log2-fold change (lfc) = 0 revealed 222 DEGs, among which 135 and 87 were up- and downregulated, respectively. The MAP4K1 gene was not among the DEG list which was expected as this gene was identified based on the “corrected” gene expression in late-stage cervical cancer samples and not by comparing the expression levels of early and late-stage groups.

2.1.b. Does this method detect other markers of late cervical cancer?

Yes, we were able to find markers of late cervical cancer that were identified by a recent study by Kannan et al., 2021. Therefore, the following information were added to the discussion of the manuscript. “According to Kaplan-Meier results in a recent study, the high expression of the MAP4K1 gene was beneficial to cervical cancer patients (Kannan et al., 2021). Their research focussed on PDCD1, a gene that is most typically related to its expression on tumor-infiltrating lymphocytes. Moreover, they showed that PDCD1 significantly co-expressed with the following 15 genes, whose high expression is beneficial for cervical cancer patients; MAP4K1, ACAP1, CST7, CXCR6, GPR171, GZMH, GZMK, P2RY10, RASAL3, SH2D1A, TBC1D10C, ZNF831, GZMM, JAKMIP1, and PSTPIP1 (Kannan et al., 2021). We compared their finding to the results of our normalization method and discovered the PDCD1 gene as well as the first 12 of the 15 genes were co-expressed within the brown module.”

2.2. More generally, it is not fully clear how the analysis of multiple cancers and the use of WGCNA modules bring new specific predictive gene markers.

2.2.a. It would be important to put these findings in the context of a known progression of gene expression in these specific cancers described in the literature.

The analysis of multiple cancers demonstrated the ability of the normalization method to cluster samples by the cancer progression, both between and within the different cancer types. We agree that the addition of the study by Kannan et al., 2021 to the manuscript further enhances the results we found in cervical cancer patients.

The use of WGCNA aids in the analysis of cancer progression as WGCNA aims to find clusters (modules) of highly correlated genes that have an impact on cancer progression. The hierarchical clustering using the WGCNA co-expressed genes helped to view the cancer progression and find subtypes within multiple cancer groups that progressed separately. Therefore, the investigation by Kannan et al., 2021 has tremendous relevance for our investigation, as PDCD1 was grouped in the brown module as well as 12 additional genes that were identified as PDCD1 genes’ strongly co-expressed genes.

2.2.b. (continuation) and a more basic approach of simply detecting differentially expressed genes (DEGs) between early and late stage for each cancer type in the evaluation dataset.

Generally, a standard DGE analysis between early and late-stage cancer samples will result in a list of DEGs specific to the two groups (early and late-stage). Therefore, clustering operation of the cancer samples based on the identified DEGs will systematically subject the samples to segregate into early and late-stage clusters, which was not the aim of the investigation (kindly refer to the response to question 2.1.a). The results of the normalization method used in the manuscript were able to segregate clusters between and within a cancer based on features in late-stage cancer samples.

2.3. Lines 420-421:

“The clustering of DLBCL and liver samples in Cluster 2, could suggest that the two cancers progress in a very similar manner.”

This grouping of two cancers in the same cluster is not a sufficiently strong argument to make this far-reaching conclusion. To make a more compelling argument, it would be important to analyze and interpret specific gene signatures that are shared between progression of these two very different cancers from very different tissue environments.

Thank you for this valuable comment. We agree that claiming the two cancers progress in a similar manner based on only our analysis is not sufficient and requires strong evidence. We unfortunately could not find evidence to support our claim from literature and therefore we decided to remove the sentence and have added the following information to the discussion.

The DLBCL samples present in Cluster 1 show gene profiles that are more upregulated in comparison to the Cluster 2 DLBCL samples. In addition, it was noted that DLBCL samples in Cluster 1 showed a higher number of extranodal sites involvement (*≥ 2*), while those in Cluster 2 showed no or low number of extranodal sites involvement (≤ 2). Common sites of extranodal spread are lung, liver, kidney, and bone marrow (Jamil and Mukkamalla, 2022). It has also been reported that DLBCL can be involved in virtually any organ (Beham-Schmid, 2017). Therefore, the DLBCL Cluster 2 found grouped with liver samples is an interesting finding, given the high prevalence of secondary liver involvement by lymphoma including DLBCL and indicates advanced disease (Rajesh et al., 2015). However, this information of secondary liver involvement in the metadata associated to DLBCL is unavailable, and requires further investigation to support the claim that DLBCL patients have liver infection, as well as the use of a higher sample number, which was not possible for this study since the public data was not available.

2.4. Conclusions about a clinically relevant clustering of samples from different subtypes of testicular cancer (lines 448-451):

Could these and similar conclusions about grouping of cancer subtypes be achieved by a more basic DEG analysis and clustering these samples within each cancer type by the expression of DEGs?

A DGE analysis would fail to segregate clusters between and within a cancer based on features in late-stage cancer samples, such as in testicular cancer. A DGE analysis between early and late-stage cancer samples will result in a list of DEGs specific to the two groups (early and late-stage). Therefore, clustering operation of the cancer samples based on the identified DEGs will systematically subject the samples to segregate into early and late-stage clusters, which was not the aim of the investigation (kindly refer to the responses to questions 2.1a and 2.2.b).

2.5. The rationale behind the choice of a relatively limited evaluation dataset was not fully described. Lines 108-110:

“The datasets examined were selected based on cancers known to have an increased survival risk among patients due to associated autoimmune diseases.”

The numbers of these patient samples (Table 1) are relatively small.

Why this selection of cancer types was made? Would autoimmune signatures contribute as an unrelated confounding factor to late-stage cancer expression signatures?

The project discussed in the context of this manuscript was conducted concurrently with another project that is focussing on Diffuse Large-B cell Lymphoma (DLBCL) samples.

It is well-known in literature that human immunodeficiency virus (HIV) increases the risk of lymphoma and autoimmune disorders can occur in patients with HIV infection. This is of great interest to our research group, as we are situated in South Africa, where the country has the highest HIV prevalence rate in the world.

Therefore, the manuscript focused on cancers known to have an increased survival risk among patients due to associated autoimmune diseases. We intend on applying this normalization method to our DLBCL projects’ samples and investigate subtypes between HIV-positive and HIV-negative DLBCL samples.

2.6. All conclusions about functional gene enrichment in detected WGCNA modules are based on the WikiPathway database. WikiPathway is only one of the public pathway databases, and arguably not the best one. It would be beneficial to analyze the enrichment of pathways from other more established resources, e.g. MSigDB, KEGG, Reactome, ChEA gene categories, as well as TF LOF and TF perturbation gene collection available from EnrichR.

The manuscript focused on protein-coding genes, and upon comparing the primary pathway databases, it was found that Reactome (v60) and KEGG (release 81) are the two largest primary databases and annotate 10 347 and 6886 unique protein-coding genes, respectively (Rahmati et al, 2019). Despite their size, they cover only about half of the human proteins (Rahmati et al. 2017). The same was found for WikiPathways that covered 50% (11 210 genes) of the protein-coding genes (Slenter et al, 2018). It was also found that the development of integrated databases remains to lack pathway annotation for about 37% of the human protein-coding genes (Rahmati et al., 2017).

Furthermore, Enrichr along with a number of R packages and online tools that perform enrichment analysis have incorporated WikiPathways into their methods and vignette examples. Human and mouse WikiPathways gene sets are now also available in the MSigDb for the GSEA software. WikiPathways are therefore used by many public databases. An additional advantage of the WikiPathways database is that the biological pathway models are collected and curated by the research community (Martens et al., 2021).

Therefore, the use of the WikiPathway database was sufficient in providing the knowledge we required in the context of this specific investigation. We were able to determine if the genes that were identified within a WGCNA module were based on tissue or cancer specific pathways.

2.7. Although some of the pathways in Fig. 6 are general pan-cancer gene sets, e.g. pathways related to cell cycle or DNA damage, some of these pathways correspond to specific cancers (gastric cancers, breast cancer, etc) that are unrelated to the analyzed cancer types. This inconsistency and specific genes detected in these unrelated cancer pathways need to be addressed.

The unrelated cancer pathways were detected in the Turquoise module which was the largest module comprising 139 genes. The unrelated cancer pathways were identified as Retinoblastoma, Gastric cancer, and breast cancer.

A study by Salarikia et al., 2022 reported that in a gene-disease network, it was indicated that diseases such as liver carcinoma, breast carcinoma, liver fibrosis, prostate cancer, ovarian carcinoma, and lung cancer were correlated with gastric adenocarcinoma through specific genes. Among the list of genes that were enriched for the gastric cancer pathway included the AURKA gene, which was also identified in the breast cancer pathway. The expression of the AURKA gene increases in many cancer cells including stomach, liver, colorectal, ovarian, breast, lung, bladder, head and neck, and prostate cancer (Miralaei et al., 2021). Other genes that were enriched included MCM4, TOP2A and RFC4 genes were also identified in the retinoblastoma pathway. MCM4 is known to be overexpressed in liver cancer (Zheng et al., 2021), TOP2A promotes lung cancer (Kou et al., 2020), RFC4 has a high expression in liver cancer, lung cancer, prostate cancer, colon cancer, brain cancers, cervical cancer, and leukemia (Li et al., 2018).

Furthermore, a study by Marshall et al., 2019 reported that many cancers have mutations in the RB pathway. There is also a close connection between the Rb pathway and cancer progression (Du and Searle, 2009, Marshall et al., 2019). The CHEK1 gene identified in the retinoblastoma pathway was also identified by the breast cancer pathway. The overexpression of CHEK1 is an important mechanism leading to the development of human malignant tumors, such as lung, bladder, colon, stomach, ovarian, and cervical cancers (Wu et al., 2019).

It can also be noted that a breast cancer pathway was discovered in a study by Ling et al., 2020 (Figure 4) that focused on gastric cancer. Therefore, when focusing on specific cancers, it is not uncommon for other cancer pathways to be identified.

A summary of all this information has been added to the revised manuscript with track changes. Please see lines 432–453.

2.8. Minor comments:

2.8.1. Lines 126-127: Xena database provides not read counts but TPM values. x should be noted as TPM.

All five datasets investigated from Xena were RNA-Seq – HTSeq – Counts, where the units were specified as log2(count+1).

2.8.2. Text labels in Figure 2,3,5 are not legible.

Figure 2, 3, and 5 have been enhanced and the resolution have been increased to the best of our abilities. We can confirm that all the figures is at a 300 pixels/inch (DPI).

Furthermore, based on a comment from another reviewer it was suggested that we included Figure 2 and 5 as supplementary figures. Therefore, the figures were moved to the supplementary section, and the numbering of Figure 2, 3, and 5 has changed in the revised manuscript to S1 Fig, Fig 2, and S2 Fig, respectively.

2.8.3. Language needs to be clarified in a few passage throughout the text, e.g. lines 425-426:

“the average overall survival rate of patients with cervical cancer represented in Cluster 5 lived longer than the patients with cervical cancer in Cluster 6”

The sentence has been amended as follows: “the average overall survival of patients with cervical cancer represented in Cluster 5 were greater than the average overall survival of cervical cancer patients in Cluster 6”

2.8.4. Heatmaps and color bars in Fig. 3,4,7: Coloring modules by bars and labeling these modules in text by the same color name is redundant. It would be more informative to have at least a very general and brief text labels by the functions of these modules.

In our opinion it is important to identify the colors, since it could be subjected to different interpretation. Also, the text color labels provide ease of reference to the wording in the article. Therefore, we have decided to keep the text with the colors.

References:

1. Beham-Schmid C. Aggressive lymphoma 2016: revision of the WHO classification. Memo. 2017;10(4):248-254. doi: 10.1007/s12254-017-0367-8. Epub 2017 Nov 30. PMID: 29250206; PMCID: PMC5725508.

2. Du W, Searle JS. The rb pathway and cancer therapeutics. Curr Drug Targets. 2009 Jul;10(7):581-9. doi: 10.2174/138945009788680392. PMID: 19601762; PMCID: PMC3151466.

3. GTEx Consortium. Genetic effects on gene expression across human tissues. Nature. 2017 Oct 11;550(7675):204-213. doi: 10.1038/nature24277. Erratum in: Nature. 2017 Dec 20;: PMID: 29022597; PMCID: PMC5776756.

4. Jamil A, Mukkamalla SKR. Lymphoma. 2022 Jul 18. In: StatPearls [Internet]. Treasure Island (FL): StatPearls Publishing; 2022 Jan–. PMID: 32809661.

5. Kannan S, O'Connor GM, Bakker EY. Molecular Mechanisms of PD-1 and PD-L1 Activity on a Pan-Cancer Basis: A Bioinformatic Exploratory Study. Int J Mol Sci. 2021 May 22;22(11):5478. doi: 10.3390/ijms22115478. PMID: 34067485; PMCID: PMC8196980.

6. Kou F, Sun H, Wu L, Li B, Zhang B, Wang X, Yang L. TOP2A Promotes Lung Adenocarcinoma Cells' Malignant Progression and Predicts Poor Prognosis in Lung Adenocarcinoma. J Cancer. 2020 Feb 10;11(9):2496-2508. doi: 10.7150/jca.41415. PMID: 32201520; PMCID: PMC7066024.

7. Li Y, Gan S, Ren L, Yuan L, Liu J, Wang W et al. Multifaceted regulation and functions of replication factor C family in human cancers. Am J Cancer Res. 2018 Aug 1;8(8):1343-1355. PMID: 30210909; PMCID: PMC6129478.

8. Ling Y, Watanabe Y, Nagahashi M, Shimada Y, Ichikawa H, Wakai T, et al. Genetic profiling for diffuse type and genomically stable subtypes in gastric cancer. Computational and Structural Biotechnology Journal. 2020 ;18:3301-3308. DOI: 10.1016/j.csbj.2020.10.021. PMID: 33240471; PMCID: PMC7666323.

9. Martens M, Ammar A, Riutta A, Waagmeester A, Slenter DN, Hanspers K, et al. WikiPathways: connecting communities. Nucleic Acids Research. 2021 Jan 8;49(D1):D613-D621. doi: 10.1093/nar/gkaa1024. PMID: 33211851; PMCID: PMC7779061.

10. Marshall AE, Roes MV, Passos DT, DeWeerd MC, Chaikovsky AC, Sage J, et al. RB1 Deletion in Retinoblastoma Protein Pathway-Disrupted Cells Results in DNA Damage and Cancer Progression. Mol Cell Biol. 2019 Jul 29;39(16):e00105-19. doi: 10.1128/MCB.00105-19. PMID: 31138663; PMCID: PMC6664603.

11. Miralaei N, Majd A, Ghaedi K, Peymani M, Safaei M. Integrated pan-cancer of AURKA expression and drug sensitivity analysis reveals increased expression of AURKA is responsible for drug resistance. Cancer Med. 2021 Sep;10(18):6428-6441. doi: 10.1002/cam4.4161. Epub 2021 Aug 1. PMID: 34337875; PMCID: PMC8446408.

12. Rahmati S, Abovsky M, Pastrello C, Jurisica I. pathDIP: an annotated resource for known and predicted human gene-pathway associations and pathway enrichment analysis. Nucleic Acids Research. 2017 Jan 4;45(D1):D419-D426. doi: 10.1093/nar/gkw1082. Epub 2016 Nov 28. PMID: 27899558; PMCID: PMC5210562.

13. Rahmati S, Pastrello C, Rossos AEM, Jurisica I. Two decades of biological pathway databases: results and challenges. In: Encyclopedia of bioinformatics and computational biology. Elsevier, New Yor,. 2019 pp 1071–1084.

14. Rajesh S, Bansal K, Sureka B, Patidar Y, Bihari C, Arora A. The imaging conundrum of hepatic lymphoma revisited. Insights Imaging. 2015 Dec;6(6):679-92. doi: 10.1007/s13244-015-0437-6. Epub 2015 Oct 6. PMID: 26443451; PMCID: PMC4656246.

15. Salarikia SR, Kashkooli M, Taghipour MJ, Malekpour M, Negahdaripour M. Identification of hub pathways and drug candidates in gastric cancer through systems biology. Sci Rep. 2022 Jun 1;12(1):9099. doi: 10.1038/s41598-022-13052-0. PMID: 35650297; PMCID: PMC9160265.

16. Slenter DN, Kutmon M, Hanspers K, Riutta A, Windsor J, Nunes N, et al. WikiPathways: a multifaceted pathway database bridging metabolomics to other omics research. Nucleic Acids Research. 2018 Jan 4;46(D1):D661-D667. doi: 10.1093/nar/gkx1064. PMID: 29136241; PMCID: PMC5753270.

17. Wu M, Pang JS, Sun Q, Huang Y, Hou JY, Chen G, et al. The clinical significance of CHEK1 in breast cancer: a high-throughput data analysis and immunohistochemical study. Int J Clin Exp Pathol. 2019 Jan 1;12(1):1-20. PMID: 31933717; PMCID: PMC6944032.

18. Zheng R, Lai G, Li R, Hao Y, Cai L, Jia J. Increased expression of *MCM4* is associated with poor prognosis in patients with hepatocellular carcinoma. J Gastrointest Oncol. 2021 Feb;12(1):153-173. doi: 10.21037/jgo-20-574. PMID: 33708433; PMCID: PMC7944152.
